# Built environment correlates of physical activity in low- and middle-income countries: A systematic review

**DOI:** 10.1371/journal.pone.0230454

**Published:** 2020-03-17

**Authors:** Sarah Elshahat, Michael O’Rorke, Deepti Adlakha

**Affiliations:** 1 School of Medicine, Dentistry and Biomedical Sciences, Centre for Public Health, Queen’s University Belfast, Belfast, Northern Ireland, United Kingdom; 2 Department of Epidemiology, College of Public Health, University of Iowa, Iowa City, Iowa, United States of America; 3 School of Natural and Built Environment, Queen’s University Belfast, Belfast, Northern Ireland, United Kingdom; Chiang Mai University Faculty of Medicine, THAILAND

## Abstract

Insufficient physical activity (PA) is the fourth major risk factor for many non-communicable diseases and premature mortality worldwide. Features of the built environment (BE) play a considerable role in determining population PA behaviors. The majority of evidence for PA-BE relationships comes from high-income countries and may not be generalizable to low- and middle-income countries (LMICs). We aim to systematically review the literature and assess the associations between perceived and/or objective BE characteristics and PA domains in LMICs. This review adopted a systematic search strategy for English language articles published between January 2000 and June 2019 from four electronic databases—Medline, Embase, Web of Science and PubMed—adhering to the PRISMA guidelines. Studies addressing the associations between self-reported and/or objective BE and PA were only included if they were conducted in LMICs, according to the World Bank classification list. Articles investigating PA-BE relationships across any age groups were included, and all study designs were eligible, except for qualitative studies and reviews. Thirty-three studies were included for evidence synthesis. Cross-sectional studies were the most prevailing study design (97%), revealing a notable gap in longitudinal PA-BE research in LMICs. A majority of the BE factors were not associated with different PA domains while others (e.g., density, proximity to services, aesthetics) exhibited an inconsistent association. Land-use mix diversity was positively associated with transport PA and the presence of recreation facilities resulted in an increase in PA during leisure-time. Increased safety from crime at night consistently increased total PA and walking levels. Research exploring the associations between BE attributes and PA behaviors in LMICs appears to be limited and is primarily cross-sectional. Longitudinal research studies with objective measures are needed for inferring well-grounded PA-BE causal relationships and informing the design of evidence-based environmental interventions for increasing PA levels in LMICs.

## 1. Introduction

Physical inactivity is a global public health issue and the fourth leading risk factor for many non-communicable diseases (NCDs), including obesity, diabetes, cardiovascular disease, stroke and some cancers [1]. The global health care costs of physical inactivity have been estimated to exceed international $53 billion annually [2]. Worldwide, about 3.2 million deaths are attributed to insufficient physical activity (PA) annually, with low- and middle-income countries (LMICs) carrying a disproportionate share of the disease burden [3]. Approximately 82% of the global NCDs deaths take place in LMICs, where the risk of premature NCD mortality is 1.5 times higher than high-income country populations [4]. Regular physical activity directly contributes to achieving the UN Sustainable Development Goal 3 (good health and well-being) as it helps prevent and treat NCDs [5]. Recognizing the importance of PA for NCD prevention, the WHO member states made an agreement to jointly work towards a relative decline in the global prevalence of inadequate PA by 10% by 2025 [6]. However, a recent study of 168 countries by Guthold et al. [7] did not reveal a significant decrease in the worldwide prevalence of insufficient PA between 2001 and 2016, (28.5 *vs* 27.5%, respectively), indicating slow progress towards meeting the global target and highlighting an urgent need for accelerated actions and policy changes for reversing these trends.

Characteristics of the built environment (BE) (human-made physical parts of the environment where people live and work, e.g., homes, buildings, streets, parks, open spaces, and transport infrastructure) play a vital role in determining individuals’ PA behaviors across four separate life-domains: domestic or home-based PA, occupational or work-related PA, travel PA, and leisure PA. Examples of the BE attributes include residential density, diversity and access to a mix of land uses and public services (e.g. parks, greenways, transit stops), pleasant neighborhood surroundings (e.g. trees, shrubs, rivers), as well as crime and traffic safety [8]. For example, accessible public parks and recreational amenities provide settings for leisure-time PA for families, individuals and institutions [9]. Availability of high-quality infrastructure for walking and cycling (e.g. sidewalks, bike paths, crosswalks) promote travel PA amongst populations [9].

Research investigating the associations between PA and BE attributes has increased rapidly over the last two decades and has become an international priority [10,11]. The vast majority of evidence in this area has primarily been limited to developed countries in Europe, North America, and Australia, with recent evidence from some LMICs in Asia, Africa and South America [8,12–14]. For instance, a systematic review of 70 studies from Europe revealed a consistent evidence for positive associations between accessible public services and PA, and reported a gap in knowledge of the BE correlates across different PA domains [15]. Similarly, Smith et al. systematically reviewed 28 studies from the US and Australia, and reported significant positive relationships between destination accessibility, travel and total PA [1]. A recent systematic review of 25 studies by Zhang et al. [16] investigated the impact of BE characteristics in parks in North America, Australia, and Europe, and reported significant positive associations between park-based leisure PA, park lighting and walking and cycling trails. However, these findings may not generalize to LMIC contexts, where BE attributes are distinct from developed countries with respect to patterns of land-use, density, design and urbanization [17]. Population growth and economic development have spurred rapid absolute increases in the number of motor vehicles in LMICs, with marked shifts in travel patterns that are moving away from public transport, walking and cycling, to private motorized vehicles [12]. Rapid, unplanned urban expansion has contributed to environmental and health hazards, including road traffic casualties, air pollution, and diminished safe spaces for walking and cycling [18]. In the last decade, Latin America has witnessed major urban changes such as an increase in utilities infrastructure and expansion in transport networks, influencing lifestyle changes and PA behaviors in populations [19,20]. For example, studies showed that residential density and street connectivity were associated with reduced likelihood of PA in urban Brazil and Colombia, respectively. [21,22]. These socio-ecological issues are associated with reduced PA levels and sedentary lifestyles, making populations of LMICs highly predisposed to NCDs and premature mortality [23].

Despite the well-documented benefits of PA, there is a gap in the current knowledge of the pathways through which the BE can promote or constrain PA across different life domains (e.g., home, work, travel and leisure) in LMICs populations. Previous reviews on this topic have been constrained by several limitations including challenges in summarising studies given their heterogeneity in terms of study design and quality and approach to evidence synthesis [24,25]. For instance, Day [24] conducted a review to investigate the impact of different BE features (e.g. density, availability of parks) and non-BE factors (e.g. weather, stray animals) on PA levels in developing countries. However, the review was narrative in nature, was not restricted to LMICs, and did not follow PRISMA guidelines or assess the quality of included studies.

The present systematic review aims to more robustly investigate the associations between BE characteristics and PA domains amongst different populations exclusively from LMICs, in order to inform and direct future research and necessary policy change towards sustainable design of the BE and to foster and promote more physically active populations in these developing regions.

## 2. Methodology

This systematic review was registered in the International Prospective Register of Systematic Reviews (PROSPERO) (Registration no. CRD42019141945) [26].

### 2.1. Search strategy

This review was conducted following the PRISMA guidelines for reporting of systematic reviews [27] (S1 Appendix). Various search terms and phrases were selected to reflect the key concepts of the review (PA, BE, LMICs), and combined using Boolean operators AND/OR (S2 Appendix). Four electronic databases were searched: Medline, Embase, Web of Science and PubMed. The search was limited to human studies and restricted to English Language papers published between January 2000 and June 2019 to summarize contemporaneous literature in the past two decades. A manual search of the reference lists of potentially relevant articles was also conducted.

### 2.2. Study selection

**Fig 1:**
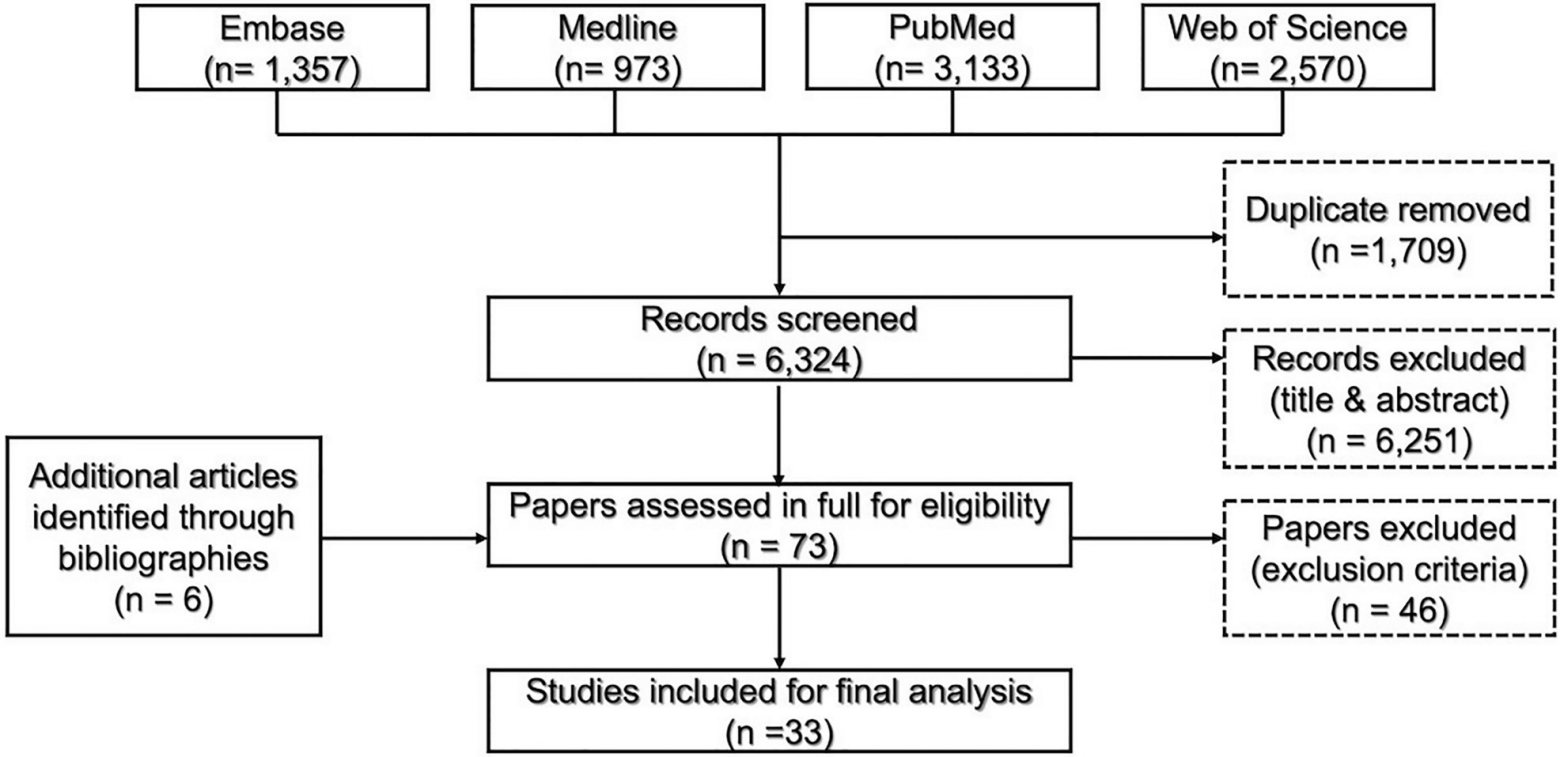
PRISMA flow chart of the research results.

Studies were considered for inclusion only if they were conducted in LMICs, according to the World Bank classification list [28]. The included studies investigated the association between objectively and/or subjectively measured BE attributes and PA. All PA domains across all age groups were included. All study designs were eligible, with the exception of qualitative studies and any types of reviews. Studies investigating the impact of PA on health outcomes without any BE attributes were excluded. A two-step procedure was adopted for selection of eligible articles from among the retrieved results. Firstly, all authors (SE, MOR, DA) screened titles and abstracts of the retrieved articles after duplicate removal. Secondly, all potentially relevant studies were re-assessed in full, applying the previously established inclusion/exclusion criteria (Fig 1). Disagreements were resolved via discussion.

### 2.3. Data extraction

A pre-defined data collection form was used for data extraction. This comprised authorship, study origin (country), characteristics of study subjects, study design, sampling methods, covariates, PA and BE variables, as well as key findings (S3 Appendix). Data was classified according to the PA domains, namely, leisure-time PA, travel PA, leisure-time walking, transport-related walking, transport-related cycling, and total PA (S4 Appendix). For each PA domain, BE characteristics were categorized into six categories according to the Neighborhood Environment Walkability Scale [29]: land-use mix diversity, density, land-use mix access, aesthetics, infrastructure for walking and cycling, as well as crime and traffic safety. Additional categories for addressing associations with income and urbanicity (degree to which a given geographical area is urban) were added.

### 2.4. Quality assessment and evidence synthesis

An eight-item checklist was constructed for assessing the quality of the included studies (S5 Appendix). Six items were adapted from both the Center for Evidence-Based Management quality appraisal guidelines, and a previously developed checklist by Barnett et al. [30,31]. These included: [1] study design [weight: cross-sectional or case study = 1, longitudinal or quasi design = 2], [2] reliable response rate (≥80%) [yes = 1, no = 0], [3] stratification of recruitment areas by suitable environmental characteristics to maintain generalizability [yes = 1, no = 0], [4] controlling for socio-demographic confounders [yes = 1, no = 0], [5] calculation of confidence interval for main results [yes = 1, no = 0], and [6] assessment of statistical significance (*p* value) [yes = 1, no = 0]. In order to improve the appropriateness of appraising the studies included in the current review, two additional items were considered; [7] method of PA measurement (weight: subjective = 0.5, objective = 1), and [8] method of BE measurement (weight: subjective = 0.5, objective = 1). Higher scorings indicate better quality: ≤4 (low quality), 4.1–5.9 (intermediate quality), 6–6.9 (high quality), and ≥7 (very high quality). The review adapted the criteria developed by Sallis et al. [32] for synthesizing evidence in relation to PA-BE associations.

## 3. Results

### 3.1. Study characteristics and quality assessment

The literature search yielded a total of 6,324 articles. In addition, six papers were identified from the bibliographies of relevant studies. Of these, only 33 articles met the eligibility criteria and were included in the final analysis (Fig 1). Most of these studies were carried out in Brazil (43%), followed by both Colombia (9%) and India (9%) (S1 Table). Approximately 94% of the included studies adopted a cross-sectional design.

Most studies were considered to be of high quality (55%), and about 30% were of moderate quality (S5 Appendix). Potential socio-demographic confounders (e.g., age, gender, socioeconomic status) were controlled for in 88% of the included studies. Only 27% of the eligible articles reported a reliable response rate (≥80%). Only 9% of the studies measured PA objectively (i.e., using an accelerometer), whereas 42% considered assessing BE attributes through objective methods (i.e., geographical information systems (GIS)). Leisure PA was the most commonly investigated PA domain across studies (48%), followed by leisure walking (30%) (S1 Table). Transport cycling was only investigated in four studies [21,33–35]. The most predominant BE attributes were safety from traffic and crime safety (45% each). We classified the results into four main subsections according to PA domains: leisure PA, travel PA, walking and cycling, as well as total PA.

### 3.2. Leisure PA

#### 3.2.1. Land-use mix diversity

Two studies showed that land-use mix diversity was not significantly related to self-reported leisure PA (Table 1).

**Table 1:** Relationships between leisure PA and BE attributes across included studies.

BE attributes	PA-BE relationships			% studies supporting the predicted association ^a^^b^	Summary codes ^c^
	Significant (+)	Significant (-)	Non-significant		
Land-use mix diversity	N/A	N/A	[8,36]	0/2 = 0%	0
Residential density	N/A	[8]	[37]	1/2 = 50%	?
Park density	[19]	N/A	[38]	1/2 = 50%	?
Leisure amenities availability	[39–42 ^d^,^43^]	[44] ^d^	N/A	4/4 = 100%	++
Land-use mix access	N/A	N/A	[8]	0/1 = 0%	0
Leisure facilities proximity	[39,45]	[46] ^e^	[36,37, 42 ^d^,^43^]	2/5 = 40%	?
Transit stops proximity	N/A	N/A	[19,36,38]	0/3 = 0%	00
Services proximity	N/A	N/A	[33,41]	0/2 = 0%	0
Aesthetics	[36,37,39]	N/A	[8,41,46]	3/6 = 50%	?
Infrastructure to walk and cycle	N/A	N/A	[8,36,41, 42 ^d^]	0/3 = 0%	00
Walkability	[47]	N/A	N/A	1/1 = 100%	+
Street connectivity	N/A	N/A	[8,44 ^d^]	0/1 = 0%	0
Sidewalks	[33]	[46] ^e^	[41,44 ^d^]	1/2 = 50%	?
Terrain slope	N/A	[19]		1/1 = 100%	-
Bike path availability	N/A		[19,41]	0/2 = 0%	0
Crime safety	[37,44 ^d^^f^]	[38] ^f^	[8,33,36,41,46 ^f^,^48^ ^f^]	1/5 = 20%	000
Traffic safety	N/A	N/A	[8,33,36,41,44 ^d^,^46^ ^f^]	0/4 = 0%	00
District income	[43,47]	N/A	N/A	2/2 = 100%	+

^**a**^ Number of articles supporting the predicted association divided by the entire number of studies investigated each BE variable.

^**b**^ Studies investigating variables signed ^**d**^, ^**e**^, or ^**f**^ was not considered in the coding unless all studies solely investigated the same variable.

^**c**^ The principles of evidence summary coding were adapted from Sallis et al [32]; +/- = positive or negative association (60–100% of articles supporting the predicted association); 0 = No relationship (0–33% of articles supporting the predicted association); ? = inconsistent relationship (34–59% of articles supporting the predicted association). Single signed codes (+, - or 0) were given for BE variables that were investigated only by 1–2 studies with respect to certain PA domains; When relationships were investigated in (3–4) or > 4 studies, double (++, - or 00) and triple (+++, - or 000) signed summary coding was applied, respectively.

^**d**^ association with physical inactivity.

^**e**^ reduced/lack of access.

^**f**^ feeling unsafe.

A cross-sectional study by Adlakha et al. [8] did not find any significant associations between perceived land-use mix diversity and self-reported leisure PA among urban Indian adults. Likewise, Jáuregui et al. [36] did not detect any significant relationships between both in urban Mexico (Cuernavaca).

#### 3.2.2. Density

Perceived residential density showed an inconsistent association with self-reported leisure PA (Table 1). A significant negative association between both was found in urban India by Adlakha et al. [8], whereas, a study by Rech et al. [37] in urban Brazil did not detect any significant relationships. Availability of recreational amenities (e.g. parks, fitness centers) was a consistently positive feature (100%) of leisure PA across LMICs (Table 1). Amorim et al [44], also showed that reduced perception of green spaces was significantly associated with lower leisure PA in urban Brazil (Pelotas).

#### 3.2.3. Land-use mix access and proximity to services

A cross-sectional study by Adlakha et al. did not reveal any significant association between land-use mix access and leisure PA among urban Indian adults [8]. Studies revealed inconsistent associations between proximity of recreational amenities and leisure PA (Table 1). While studies by both Akpinar et al. [39] and Chen et al. [45] detected significant positive associations for urban green spaces proximity, Jáuregui et al. [36], Hino et al. [43] and Rech et al. [37] did find any significant relationships. Studies did not demonstrate any significant relationships between proximity of services (i.e., transit stops, stores) and leisure PA (Table 1).

#### 3.2.4. Aesthetics

Studies revealed inconsistent relationships between perceived aesthetics and self-reported leisure PA in different LMICs (Table 1). Aesthetics was a significant positive predictor in urban Turkey (Aydın), Mexico (Cuernavaca) and Brazil (Curitiba) [36,37,39], whereas it did not show any significant relationships in urban India (Chennai), Uganda (Kampala) and Brazil (Recife) [8,41,46].

#### 3.2.5. Infrastructure for walking and cycling

Studies revealed no significant relationships between leisure PA and either street connectivity or presence of infrastructure for walking and cycling (Table 1). When investigating perceived sidewalks availability, Parra et al. [33] detected a positive relationship with self-reported leisure PA, whereas, Vancampfort et al. [41] did not note any significant association. Finally, a study by Gomez et al. [19] in Colombia (Bogotá) found a significant negative association between objectively-measured land slope of ≥ 4% and self-reported leisure PA.

#### 3.2.6. Crime and traffic safety

Traffic safety was not significantly associated with leisure PA across LMICs (Table 1). Crime safety was also not related to self-reported leisure PA across studies, with the exception of one study by Rech et al. [37] who reported a significant positive association in urban Brazil.

#### 3.2.7. District income

Only two studies investigated the relationship between district income (assessed through GIS) and leisure-time PA. These showed a significant positive association between high area income and self-reported leisure PA in southern Brazil (Curitiba) [43,47].

### 3.3. Travel PA

#### 3.3.1. Land-use mix diversity

Two separate studies by Adlakha et al. [8,49] showed significant positive relationships between perceived land-use mix diversity and self-reported travel PA in urban Indian adults. Jáuregui et al [36], on the other hand, did not note any significant associations between both in urban Mexico.

#### 3.3.2. Density

While residential density predicted significantly increased odds for travel PA in India (Chennai), it was not significantly related to multi-modal or active commuting in the same Indian City [8,49].

#### 3.3.3. Land-use mix access and proximity to services

Adlakha et al. [8] reported no significant relationships between land-use mix access and travel PA in urban India (Chennai). While Adlakha et al. [49] reported a strong positive association between transit stop proximity and travel PA in urban India, a study by Jáuregui et al. [36] did not demonstrate a significant association between both in urban Mexico.

#### 3.3.4. Aesthetics

Three studies showed significant negative relationships between neighborhood aesthetics and travel PA (Table 2).

**Table 2:** Relationships between travel PA and BE attributes across included studies.

BE attributes	PA-BE relationships			% studies supporting the predicted association ^a^^b^	Summary codes ^c^
	Significant (+)	Significant (-)	Non-significant		
Land-use mix diversity	[8,49]	N/A	[36]	2/3 = 67%	++
Residential density	[8]	N/A	[49]	1/2 = 50%	?
Leisure amenities availability	N/A	N/A	[44]^d^	0/1 = 0%	0^d^
Land-use mix access	N/A	N/A	[8,49]	0/2 = 0%	0
Recreational facilities proximity	N/A	[36]	[46]	1/2 = 50%	?
Transit stops proximity	[49]	N/A	[36]	1/2 = 50%	?
Aesthetics	[46]^f^	[8,49]	[36]	2/3 = 67%	--
Infrastructure to walk/cycle	N/A	N/A	[8,36,49]	0/3 = 0%	00
Street connectivity	N/A	[8,49]	[44] ^d^	2/2 = 100%	-
Sidewalks	N/A	N/A	[44 ^d^,^46^]	0/1 = 0%	0
Crime safety	N/A	[8,49]	[36,44 ^d^,^46^ ^e^]	2/3 = 67%	--
Traffic safety	[44] ^d^	N/A	[8,36,46 ^e^,^49^]	0/3 = 0%	00

^**a**^ Number of articles supporting the predicted association divided by the entire number of studies investigated each BE variable.

^**b**^ Studies investigating variables signed ^**d**^, ^**e**^, or ^**f**^ was not considered in the coding unless all studies solely investigated the same variable.

^**c**^ The principles of evidence summary coding were adapted from Sallis et al [32]; +/- = positive or negative association (60–100% of articles supporting the predicted association); 0 = No relationship (0–33% of articles supporting the predicted association); ? = inconsistent relationship (34–59% of articles supporting the predicted association). Single signed codes (+, - or 0) were given for BE variables that were investigated only by 1–2 studies with respect to certain PA domains; When relationships were investigated in (3–4) or > 4 studies, double (++, - or 00) and triple (+++, - or 000) signed summary coding was applied, respectively.

^**d**^ association with physical inactivity.

^**e**^ feeling unsafe.

^**f**^ unpleasant aesthetics.

Aesthetics predicted significantly reduced odds of transport PA among urban Indian adults in studies by Adlakha et al. [8,49]. Hallal et al. [46] also reported an inverse relationship between aesthetics and travel PA in urban Brazil.

#### 3.3.5. Infrastructure for walking and cycling

Studies did not detect any significant relationships between presence of infrastructure for walking and bicycling and self-reported travel PA across LMICs (Table 1). Adlakha et al. [8,49] reported a significant negative association between perceived street connectivity and self-reported travel PA in India (Chennai).

#### 3.3.6. Crime and traffic safety

Perceived traffic safety was unrelated to travel PA across LMICs (Table 1). On the other hand, surveys by Adlakha et al. [8,49] showed that higher perceived crime safety in urban India was significantly negatively associated with travel PA.

### 3.4. Walking and cycling

#### 3.4.1. Density

Objectively measured residential density (GIS) predicted increased and decreased odds of transport walking and transport cycling in urban Brazil, respectively (Table 3).

**Table 3:** Relationships between walking/ cycling and BE attributes across included studies.

PA type	BE attributes	PA-BE relationships			% studies supporting the predicted association ^a^^b^	Summary codes ^c^
		Significant (+)	Significant (-)	Non-significant		
**Transport walking**	Residential density	[21,50]	N/A	N/A	2/2 = 100%	+
	Commercial density	[21]	N/A	N/A	1/1 = 100%	+
	Street density	[35]	N/A	[21,50]	1/3 = 33%	?
	Leisure amenities availability	N/A	N/A	[50]	0/1 = 0%	0
	Transit stops proximity	N/A	N/A	[21]	0/1 = 0%	0
	Services proximity	[51]	N/A	[33]	1/2 = 50%	?
	Aesthetics	N/A	N/A	[51]	0/1 = 0%	0
	Infrastructure for walking	N/A	N/A	[34]	0/1 = 0%	0
	Walkability	[47]	N/A	N/A	1/1 = 100%	+
	Street connectivity	[50]	N/A	[21]	1/2 = 50%	?
	Sidewalks	[50]	N/A	[33,34]	1/3 = 33%	?
	Terrain slope	N/A	N/A	[21,33]	0/2 = 0%	0
	Paved streets	[50]	N/A		1/1 = 100%	+
	Bike path availability	N/A	N/A	[21,33]	0/2 = 0%	0
	Bike path proximity	N/A	N/A	[21]	0/1 = 0%	0
	Traffic safety	N/A	N/A	[21,33,34,51]	0/4 = 0%	00
	Total crime safety	[33]	N/A	[51]	1/2 = 50%	?
	Crime safety during day	N/A	N/A	[48] ^e^	0/1 = 0%	0 ^e^
	Crime safety at night	N/A	[48] ^e^	N/A	1/1 = 100%	- ^e^
	District income	N/A	N/A	[21,47,50]	0/3 = 0%	00
**Leisure walking**	Residential density	N/A	N/A	[50]	0/1 = 0%	0
	Street density	[50]	N/A	N/A	1/1 = 100%	+
	Leisure amenities availability	[43]	N/A	[50]	1/2 = 50%	?
	Recreational facilities proximity	[43]	N/A	[14,36,46]	1/4 = 25%	00
	Transit stops proximity	N/A	N/A	[36]	0/1 = 0%	0
	Services proximity	[51]	N/A	[33]	1/2 = 50%	?
	Squares proximity	N/A	N/A	[14]	0/1 = 0%	0
	Aesthetics	[36]	N/A	[46,51]	1/3 = 33%	00
	Infrastructure for safe walking	N/A	N/A	[20,36]	0/2 = 0%	0
	Walkability	N/A	N/A	[47]	0/1 = 0%	0
	Street connectivity	N/A	N/A	[50]	0/1 = 0%	0
	Sidewalks	[20] ^d^	[46] ^d^	[33,50]	0/2 = 0%	0
	Paved streets	N/A	N/A	[50]	0/1 = 0%	0
	Bike path proximity	[14]	N/A	N/A	1/1 = 100%	+
	Crime safety	N/A	N/A	[33,36,46,48 ^e^,^51^]	0/4 = 0%	00
	Traffic safety	[36]	N/A	[20,33,46,51]	1/5 = 20%	000
	District income	[43,50]	N/A	[47]	2/3 = 67%	++
**Total walking**	Residential density	N/A	N/A	[12]	0/1 = 0%	0
	Park density	[22]	N/A		1/1 = 100%	+
	Leisure amenities availability	N/A	N/A	[12,41]	0/2 = 0%	0
	Transit stops proximity	N/A	N/A	[12,22]	0/2 = 0%	0
	Services proximity	[41]	N/A	[12]	1/2 = 50%	?
	Aesthetics	[12]	N/A	[41]	1/2 = 50%	?
	Infrastructure for safe walking	N/A	N/A	[41]	0/1 = 0%	0
	Street connectivity	N/A	[22]	[12]	1/2 = 50%	?
	Sidewalks	[41]	N/A	[12,22]	1/3 = 33%	00
	Terrain slope	N/A	[22]	N/A	1/1 = 100%	-
	Bike path availability	N/A	N/A	[12]	0/1 = 0%	0
	Bike path proximity	N/A	N/A	[41]	0/1 = 0%	0
	Crime safety during day	[52]	N/A	[12,41]	1/3 = 33%	00
	Crime safety at night	[12,52]	N/A	[41]	2/3 = 67%	++
	Traffic safety	[22]	N/A	[12,41,52]	1/4 = 25%	00
**Transport cycling**	Residential density	N/A	[21]	N/A	1/1 = 100%	-
	Commercial density	N/A	N/A	[21]	0/1 = 0%	0
	Street density	[35]	N/A	[21]	1/2 = 50%	?
	Transit stops proximity	N/A	N/A	[21]	0/1 = 0%	0
	Services proximity	[33]	N/A	N/A	1/1 = 100%	+
	Infrastructure for cycling	N/A	N/A	[34]	0/1 = 0%	0
	Street connectivity	N/A	N/A	[21]	0/1 = 0%	0
	Sidewalks	N/A	N/A	[33,34]	0/2 = 0%	0
	Terrain slope	N/A	N/A	[21,33]	0/2 = 0%	0
	Bike path availability	N/A	N/A	[21,33]	0/2 = 0%	0
	Bike path proximity	N/A	N/A	[21]	0/1 = 0%	0
	Crime safety	N/A	N/A	[33]	0/1 = 0%	0
	Traffic safety	N/A	[21]	[33,34]	1/3 = 33%	00
	District income	N/A	[21]	N/A	1/1 = 100%	-

^**a**^ Number of articles supporting the predicted association divided by the entire number of studies investigated each BE variable.

^**b**^ Studies investigating variables signed ^**d**^, or ^**e**^ was not considered in the coding unless all studies solely investigated the same variable.

^**c**^ The principles of evidence summary coding were adapted from Sallis et al [32]; +/- = positive or negative association (60–100% of articles supporting the predicted association); 0 = No relationship (0–33% of articles supporting the predicted association); ? = inconsistent relationship (34–59% of articles supporting the predicted association). Single signed codes (+, - or 0) were given for BE variables that were investigated only by 1–2 studies with respect to certain PA domains; When relationships were investigated in (3–4) or > 4 studies, double (++, - or 00) and triple (+++, - or 000) signed summary coding was applied, respectively.

^**d**^ reduced/lack of access.

^**e**^ feeling unsafe.

Hino et al. [21] reported that commercial density in Brazil (Curitiba) was significantly positively associated with self-reported transport walking, but it was not significantly related to transport cycling. Street density was a significant positive predictor for leisure walking in urban Brazil [50], yet it showed inconsistent associations with both transport walking and cycling across LMICs (Table 1). When combining both transport and leisure walking, Gomez et al. [22] found a positive relationship with objectively assessed park density (GIS) in urban Columbia.

#### 3.4.2. Land-use mix access and proximity to services

Studies did not reveal any significant relationships between proximity to transit stops and leisure walking, transport walking or transport cycling (Table 1). Perceived proximity to public services (e.g. stores, parks and transport stations) in suburban China (Shanghai) was associated with significantly increased odds for both transport and leisure walking [51]. Parra et al. [33], however, did not show significant relationships between perceived presence of public services with a 10-min walk in urban Brazil and either transport or leisure walking, yet a significant positive association was found for transport cycling.

#### 3.4.3. Aesthetics

Perceived aesthetics was not significantly related to either leisure or transport walking across LMICs (Table 1). Jia et al. [51] did not detect any significant relationships between aesthetics and either self-reported transport or leisure walking in urban Chinese adults. A survey by Hallal et al. [46] also did not reveal any significant associations between perceived presence of pleasant surroundings and leisure walking in urban Brazil.

#### 3.4.4. Infrastructure for walking and cycling

Studies did not detect any significant relationships for presence of infrastructure for walking and cycling and leisure walking, transport walking or transport cycling (Table 1). Giehl et al. [50], however, reported significant positive associations between transport walking and street connectivity, sidewalks or paved streets (assessed through GIS) among Brazilian older adults. When examining the impact of lack of sidewalks on self-reported leisure walking among urban Brazilian adults, Hallal et al. [46] reported a negative association, whereas Gomes et al. [20] noted a positive relationship. Bike path existence within 500-m buffers in Brazil (Sao Paulo) was associated with significantly increased odds for leisure walking in a study by Florindo et al. [14]. On the other hand, terrain slope of ≥5% and high street connectivity (measured using GIS) predicted reduced odds of total walking among urban Columbian adults [22].

#### 3.4.5. Crime and traffic safety

Studies did not show significant associations between traffic safety and leisure walking, transport walking or transport cycling across LMICs (Table 1). While perceived crime-related safety did not exhibit any association with either leisure walking or transport cycling, it was a significant positive predictor for self-reported transport walking in Brazil (Curitiba) [33]. When combining both transport and leisure walking, Oyeyemi et al. [52] noted significant positive associations with both perceived crime safety during day and at night.

#### 3.4.6. District income

Studies did not show any significant associations between district income and transport walking (Table 1). On the other hand, objectively assessed medium and high area income in Brazil predicted significantly increased odds of self-reported leisure walking in Curitiba and Florianópolis, respectively [43,50]. In contrast, high district income was inversely associated with transport cycling among urban Brazilian adults [21].

### 3.5. Total PA

#### 3.5.1. Density

A study in Nigerian students (University of Ibadan) by Oyeyemi et al. [12] did not reveal any significant association between perceived residential density or existence of leisure facilities and total PA (Table 4).

**Table 4:** Relationships between total PA and BE attributes across included studies.

BE attributes	PA-BE relationships			% studies supporting the predicted association ^a^^b^	Summary codes ^c^
	Significant (+)	Significant (-)	Non-significant		
Residential density	N/A	N/A	[12]	0/1 = 0%	0
Leisure amenities availability	N/A	N/A	[12]	0/1 = 0%	0
Recreational facilities proximity	[53]	N/A	N/A	1/1 = 100%	+
Transit stops proximity	[12]	[53]	[54]	1/3 = 33%	00
Services proximity	N/A	[54]	[12,33]	1/3 = 33%	00
Aesthetics	N/A	N/A	[12,53]	0/2 = 0%	0
Infrastructure for cycling/walking	[55] ^d^	N/A	N/A	1/1 = 100%	+ ^d^
Street connectivity	N/A	[12] ^e^	N/A	1/1 = 100%	− ^e^
Sidewalks	[33]	N/A	[12,53]	1/3 = 33%	00
Terrain slope	[33]	N/A		1/1 = 100%	+
Bike path availability	[33]	N/A	[12]	1/2 = 50%	?
Crime safety during day	[33,53]	[52]	[12]	2/4 = 50%	?
Crime safety at night	[33,52,53]	N/A	[12]	3/4 = 75%	++
Traffic safety	N/A	[12]	[33,53]	1/3 = 33%	00
Urbanicity	[56–58]^d^	N/A	N/A	3/3 = 100%	++ ^d^

^**a**^ Number of articles supporting the predicted association divided by the entire number of studies investigated each BE variable.

^**b**^ Studies investigating variables signed ^**d**^ or ^**e**^ was not considered in the coding unless all studies solely investigated the same variable.

^**c**^ The principles of evidence summary coding were adapted from Sallis et al [32]; +/- = positive or negative association (60–100% of articles supporting the predicted association); 0 = No relationship (0–33% of articles supporting the predicted association); ? = inconsistent relationship (34–59% of articles supporting the predicted association). Single signed codes (+, - or 0) were given for BE variables that were investigated only by 1–2 studies with respect to certain PA domains; When relationships were investigated in (3–4) or > 4 studies, double (++, - or 00) and triple (+++, - or 000) signed summary coding was applied, respectively.

^**d**^ association with physical inactivity.

^**e**^ reduced/lack of access.

#### 3.5.2. Land-use mix access and proximity to services

Proximity of public services and transit stops did not exhibit any relationships with total PA (Table 1). Proximity to recreational amenities, however, significantly increased odds for total PA among urban Mexican adults [53].

#### 3.5.3. Aesthetics

Studies did not report any significant relationships between perceived aesthetics and total PA (Table 1). Jáuregui et al. [53] did not detect any significant relationship between aesthetics and objectively measured total PA (accelerometer) in urban Mexican adults. Perceived pleasant surroundings was also not related to self-reported PA in young Nigerian adults [12].

#### 3.5.4. Infrastructure for walking and cycling

Parra et al. [33] noted significant positive associations between total PA and presence of sidewalks, bike paths or steep terrain among urban Brazilian adults. Perceived lack of four-way intersections predicted significantly reduced odds of participation in total PA in Nigerian university students [12].

#### 3.5.5. Crime and traffic safety

While perceived crime safety during the day showed inconsistent relationships, crime safety at night was consistently positively associated with total PA across LMICs (Table 1). Two studies did not show any significant relationship between perceived traffic safety and total PA among urban Mexican and Brazilian adults (Table 1). Oyeyemi et al. [12] however, reported a significant unexpected negative association between perceived traffic safety and self-reported total PA (excluding walking) among young Nigerian adults.

#### 3.5.6. Urbanicity

Three studies demonstrated a consistent significant positive association (100%) between urbanicity and self-reported physical inactivity (Table 1). A cross sectional analysis of data from 46 LMICs showed a significant positive association between urbanicity and not meeting the WHO PA guidelines [56]. Similarly, urbanization was a significant positive predictor for self-reported physical inactivity in separate studies by Katulanda et al. (in Sri Lanka) [57] and Allender et al. (in India) [58].

## 4. Discussion

This systematic review aimed to provide a better understanding of the impact of BE attributes on PA behaviors in LMICs populations. We identified a supportive role of the BE environment on PA domains, however, there were considerable variations in BE correlates across different PA domains in LMICs. All eligible studies included in this review were descriptive in nature (cross-sectional and case-series design), revealing the gap in longitudinal and analytical research in LMICs. More than half of included studies (58%) were conducted in Latin America (Mexico, Brazil, and Columbia), highlighting the paucity of research on this topic in other developing countries.

Perceived land-use mix diversity was not significantly related to leisure PA, however, there was a consistent positive association with travel PA across LMICs. Our findings produced limited data concerning land-use mix access and did not show any significant relationships with either leisure PA or travel PA. Similarly, in a systematic review of 64 studies from both high-income and LMICs, Cleland et al [59] revealed no significant relationship between land-use mix access and either leisure or travel PA in older adults.

Residential density was significantly positively related to transport walking, but negatively associated with transport cycling. A possible reason for this inverse relationship with cycling may be the lack of bicycling infrastructure such as segregated bike paths, specialized traffic signs, and signals and secured bike racks in highly dense residential areas [60]. In many LMICs, perceptions that roads are dangerous and uncomfortable, largely due to high volumes and high speeds of motorised traffic are well-established barriers to cycling [61]. Transport-related walking and bicycling are still common means of transportation especially in lower income groups, accounting for 50% to 70% of commuter trips in some urban areas, yet social prejudice against bicycle use prevails, and car ownership is still seen as a sign of social status [62]. Similar to our study, a survey by Forsyth et al. [63] revealed that residential density was significantly positively correlated with travel walking in the US (Minnesota). Commercial density was investigated only in one study in urban Brazil, where it predicted significantly increased odds of transport walking, yet it was not significantly related to transport cycling [21]. Across LMICs, availability of recreational amenities showed a consistent positive influence (100%) on leisure PA only. Existence of leisure amenities was also a significant positive predictor for leisure PA in Australia (Adelaide) and the US (North Carolina) [64,65].

This review showed that subjectively or objectively measured proximity to transit stops was not significantly associated with PA domains across LMICs. A meta-analysis of nine studies from North America and the UK, however, concluded that access to public transit was significantly positively correlated with participation in PA [66]. In contrast to developed countries, the lack of association between proximity to transit stops and PA in LMICs may be due to the widespread availability of alternative privately-operated ride-hailing services (e.g., minibuses, taxis, autorickshaws or tuk-tuks) providing fast connections to transit stops and minimizing PA levels [67]. Perceived aesthetics showed a significant association (negative direction) only with travel PA. In contrast, the presence of pleasant surroundings increased the likelihood of participation in PA among Japanese adults [68]. These conflicting results may be explained by discrepancies in different populations’ perceptions about aesthetics and its importance in promoting PA behavior.

Our results reveal that availability of infrastructure for safe walking and cycling was not significantly associated with any PA domains across LMICs. A study in older American adults also did not find significant relationships between perceived presence of walking and cycling facilities and self-reported total PA in seven rural areas in North Carolina [69]. Self-reported and/or objective street connectivity did not show significant associations with leisure PA, leisure walking or transport cycling, however, it was significantly inversely related to travel PA. The study findings demonstrated that presence of bike paths was not significantly related to any PA domains. The 2012 Active People Survey in England, however, showed that bike path networks were significantly positively related to self-reported cycling [70]. These variations may be explained by the juxtaposition of different means of transportation (pedestrians, bicyclists, and drivers), poor road infrastructure, lack of walking and cycling paths, weak road regulations and law enforcement in LMICs [71]. These inconsistent findings may also be related to socio-cultural differences and gendered norms in developing countries. For example, cycling is perceived as more appropriate for younger men, but an improper and unacceptable activity for women to engage in as per societal traditions in some LMICs [72].

Reviewed literature showed that perceived crime safety was not significantly related to leisure PA, yet it was significantly inversely associated with travel PA. These inconclusive results were also reported in previous studies, highlighting the need for further investigation of the influence of crime-related safety on different PA domains [73]. Perceived traffic safety was not significantly related to any PA domain across LMICs. On the other hand, perceived traffic-related safety predicted increased odds of participation in PA among Dutch adults in a study by Jongeneel-Grimen et al. [74]. These variations may be attributed to differences in perceived meaning of traffic safety among populations of different countries, where LMICs populations are more accustomed to high traffic volumes. Similar to Jongeneel-Grimen et al.’s study, a narrative review by Day [24] reported an association between traffic safety and travel PA in developing countries. However, we believe that findings from this review may have been inconclusive due to inconsistencies in evidence synthesis. Along with BE features, this review also examined non-BE factors (e.g. stray animals, weather), which may have impacted the overall study findings and conclusions.

Some studies showed that high district income was significantly positively associated with both leisure PA and leisure walking. Kari et al. [75] also reported that higher district income was a significant positive predictor for leisure PA among Finnish adults. High-income populations often have the resources to pay for memberships in private, well-maintained leisure centres, offering a wide variety of recreational activities (e.g. swimming and fitness classes, gymnasium, golf courts) [76].

Studies revealed that urbanicity significantly increased the likelihood of physical inactivity across LMICs. In both developed and developing countries, there is strong evidence that urbanicity is associated with increased sedentary behavior and diminishing levels of PA [58]. Urbanicity was also a significant positive predictor of physical inactivity among older populations in Europe [77]. Urban environment factors such as high population density, motorization, lack of safety from traffic and crime, diminishing space for walking, cycling and recreational activities, and excessive involvement in screen-based leisure time activities (e.g., television watching, video games, computer use, etc.) are associated with reduced PA and higher sedentary behaviors [23]. Measures of urbanicity have limitations as they only consider basic aspects of urbanization [78]. In LMIC contexts, these measures may not be capturing the context accurately as neighborhoods may have differing types of BE infrastructure (e.g., number of paved roads, proximity to markets, etc.), highlighting the need to create a hierarchy of BE features corresponding to levels of urbanicity for LMICs.

## 5. Strengths & limitations

This systematic review focuses on addressing the relationship between BE and PA in LMICs, which have thus far garnered limited research attention. Given that there are no gold standards for the selection of databases in the conduct of systematic reviews, we adopted a rigorous search strategy across four electronic databases including MEDLINE, PubMED, EMBASE and Web of Science. In conjunction with a robust search strategy, we feel confident that no pertinent articles were missed. This review was registered in PROSPERO to increase its transparency and minimize reporting bias. The findings were categorized by PA domains in order to allow effective reporting on the relationship between each separate domain and with BE features. This study, however, has some limitations for consideration. Given the large number of PA and BE variables examined, different statistical methods adopted by studies, and the inconsistency in the measurement of different PA domains and BE characteristics across the included studies, it was not feasible to do a meta-analysis. The majority of evidence in the current study was based on cross-sectional designs, which cannot infer causation [79]. Furthermore, more than half of the included studies relied on self-reported questionnaires for measuring BE and/or PA, which made our results subject to different types of bias (e.g. social desirability, recall bias) [80]. This review only included English publications, which posed the likelihood of excluding other relevant non-English articles from LMICs.

## 6. Conclusions and recommendations

Research investigating the relationship between BE and PA behaviors amongst LMICs populations appears to be limited. The majority of evidence in this field comes from cross-sectional studies, highlighting the gap in longitudinal, analytical and intervention research in LMICs. The majority of the investigated BE attributes were either unrelated or showed inconsistent relationships with all PA domains.

Based on the findings of this systematic review, we propose that future BE-PA research and policy making in LMICs must focus on longitudinal assessments in different geographical areas (both rural and urban), with sub-group variations (e.g., age, gender, socioeconomic status and ethnicity) in order to infer reliable, temporal BE-PA relationships that will inform public health interventions and practice. Utilizing a combination of both validated/standardized objective and self-reported measures for assessing BE attributes and different PA domains can minimize any potential information bias. Recommendations include the development of tailored environmental interventions for promoting PA amongst LMICs populations, with feasibility studies for testing, and intervention evaluation before scaling up.

## Supporting information

S1 Appendix PRISMA checklist. (DOCX)

S2 Appendix The search terms used in the searched databases. (DOCX)

S3 Appendix The standardized form with data extracted from the 33 eligible studies. (DOCX)

S4 Appendix Classification of data according to PA domains. (DOCX)

S5 Appendix Quality assessment of the included studies. (DOCX)

S1 Table Numerical distribution and characteristics of the included studies. (DOCX)
