# Supplementary material for: Built environment correlates of physical activity in low- and middle-income countries: A systematic review
Source: PLoS One. 2020 Mar 17;15(3):e0230454. doi: 10.1371/journal.pone.0230454 (PMC7077823; doi:10.1371/journal.pone.0230454)
Supplement: S2 Appendix — (DOCX) [file pone.0230454.s002.docx]

**S2 Appendix. The search terms used in the searched databases**

| 1 | Urban planning.mp. or City Planning/ |
| --- | --- |
| 2 | Environment Design/ or urban design.mp. |
| 3 | Exercise/ or Public Health/ or built environment.mp. |
| 4 | physical environment.mp. |
| 5 | liv*ab*.mp. |
| 6 | walk*.mp. or Walking/ |
| 7 | 1 or 2 or 3 or 4 or 5 or 6 |
| 8 | Cycl*/ or bicycl*.mp. |
| 9 | Physical activity.mp. or Exercise/ |
| 10 | 8 or 9 |
| 11 | ((developing or less* developed or under developed or underdeveloped or middle income or low* income) adj (economy or economies)).ti,ab. |
| 12 | ((developing or less* developed or under developed or underdeveloped or middle income or low* income or underserved or under-served or deprived or poor*) adj (countr* or nation? or population? or world)).ti,ab. |
| 13 | (low* adj (gdp or gnp or gross domestic or gross national)).ti,ab. |
| 14 | (low adj3 middle adj3 countr*).ti,ab. |
| 15 | (lmic or lmics or third world or lami countr*).ti,ab. |
| 16 | transitional countr*.ti,ab. |
| 17 | 11 or 12 or 13 or 14 or 15 or 16 |
| 18 | 7 and 10 and 17 |
| 19 | limit 18 to (humans and yr="2000 -Current") |
