# Supplementary material for: Built environment correlates of physical activity in low- and middle-income countries: A systematic review
Source: PLoS One. 2020 Mar 17;15(3):e0230454. doi: 10.1371/journal.pone.0230454 (PMC7077823; doi:10.1371/journal.pone.0230454)
Supplement: S3 Appendix — (DOCX) [file pone.0230454.s003.docx]

**S3 Appendix. The standardized form with data extracted from the 33 eligible studies**

| **Author, date** | **Title** | **Study design** | **Country** | **Study subjects** | **Sample** | **PA assessment tool, variables** | **BE assessment tool, variables** | **Covariates** | **Main findings/ associations** |
| --- | --- | --- | --- | --- | --- | --- | --- | --- | --- |
| Adlakha et al, 2017 | “Can we walk?” Environmental supports for physical activity in India | Cross-sectional | Urban India, Chennai | (N = 370), 37.9±15.3yrs, 54.2% females, 61.2% married, 62.5% employed, and 49.7% with a graduate degree | Stratified and random sampling | IPAQ-LF (Leisure & travel PA), using cut-off of 150 min/week | NEWS-India- Eight variables  (a) Residential density, (b) Diversity, (c) Land use mix-access, (d) Street connectivity, (e) Infrastructure to walk/ cycle, (f) Aesthetics, (g) Traffic safety, and (h) Crimes safety. | Age, gender, religion, SES, marital status, educational level, and employment status (adjusted for age, gender, SES) | - The study found a significant negative association between residential density and achieving ≥150 min/week MVPA for leisure (aOR=0.6, 95%CI; 0.4-1.0). - The study detected a significant positive association between residential density (aOR= 1.9, 95%CI; 1.2-3.2) and land use mix-diversity (aOR= 2.1, 95%CI; 1.2-3.6) and achieving ≥150 min/week MVPA for transportation. |
| Adlakha et al, 2018 | Exploring neighborhood environments and active commuting in Chennai, India | Cross-sectional | Urban India, Chennai | (N = 370) 37.9± 15.3 yrs, 47.2% female, 61.2% married, 82.2% Hindu, 49.7% graduate degree | Stratified and random sampling | Self-reported- questionnaire (travel-PA) | NEWS-India- Nine variables  (a) Residential density, (b) Diversity, (c) Land use mix-access, (d) Street connectivity, (e) Infrastructure to walk/ cycle, (f) Aesthetics, (g) Traffic safety, and (h) Crime safety, and (i) Transit stop proximity | Age, gender, race, marital status, education, household car ownership, and household income (adjusted for age, gender, car ownership) | - The study revealed a significant positive association between transit stop proximity (aOR =5.0, 95%CI; 1.7-14.4) and land-use mix diversity (aOR =6.8, 95%CI; 2.3-20.6) and active/multimodal commuting. - A significant negative association was found between aesthetics (aOR =0.2, 95%CI; 0.0–1.0), crime safety (aOR =0.2, 95%CI; 0.1–0.6), and street connectivity (aOR =0.2, 95%CI; 0.1–0.6), and likelihood of active/multimodal commuting. |
| Akpinar et al, 2016 | How are characteristics of urban green space (UGS) related to levels of physical activity: Examining the links | On-site, in-person interview | Aydın, Turkey, seven different urban green spaces | (N= 420), 50.12% females, 16.01% aged (25-34yrs), 64.38% married, 30.95% primary school, and 49.2% employed | Quota sampling from seven selected urban green spaces | Self-reported PA questionnaire (leisure PA in UGS)- Rating PA frequency in UGS on 5-point scale. | Self-reported questionnaire- Three variables (a)Distance to the nearest UGS, (b) Aesthetics, and (c) Recreational facilities existence. | Sex, age, marital status, education level, occupation and household income level (all adjusted) | - Significant positive association was detected between PA frequency in UGS and many trees existence (b=0.326, 95% CI; 0.105–546) and near distance to UGS (b=0.097, 95% CI; 0.004–0.197). |
| Allender et al, 2010 | Level of urbanization and non-communicable disease risk factors in Tamil Nadu, India | Cross-sectional | 7 urban areas in India | (N=3705) [response rate; 97%], 39.2± 14.4yrs, 51.2% females and 73.4% had some schooling, | Random and purposfu-l sampling | Self-reported questionnaire (total PA), using cut-off of 150 min/week | Urbanicity measure - Three variables (a) high urbanicity, (b) medium urbanicity, and (c) low urbanicity | Age, gender (adjusted) | - Physical inactivity was positively related to urbanicity amongst males (medium urbanicity- aOR=1.7, 95%CI; 1.2- 2.5; high urbanicity- aOR=3.2, 95%CI; 2.5- 4.2) and females (high urbanicity- aOR=4.2, 95%CI; 3.0- 5.7). |
| Amorim et al, 2010 | Physical activity levels according to physical and social environmental factors in a sample of adults living in South Brazil | Cross-sectional | Pelotas, South Brazil | (N=972), [ response rate; 90.7%], 57.0% females, 26.1% (20-29yrs), 82.0% white, and 47.8% with good health status | Multi-stage stratified random sampling | IPAQ-Long version: (Leisure and transport PA), using cut-off of 150 min/week | NEWS- Brazil- Fie variables  (a) Sidewalks existence, (b) Green areas existence, (c) Traffic safety (d) Cross-walks and (e) Crime existence, | Sex, age, socioeconomic level, and skin color (adjusted) | - Significant positive association between existence of crimes (aPR= 1.10; 95%CI; 1.00–1.22) and achieving < 150 min/week MVPA. - Significant negative association between existence of green areas (aPR=0.87, 95%CI; 0.81–0.94) and achieving < 150 min/week MVPA. |
| Cervero et al, 2009 | Influences of Built Environments on Walking and Cycling: Lessons from Bogotá | Cross-sectional | Bogotá, Colombi-a | (N=1000), [response rate= 66.7%] | Multi-stage stratified random sampling | IPAQ- (WT & CT), using cut-off of 30 min/day | GIS- using 500 and 1000-m buffers; Street density ≥ 0.20 or Street density < 0.20 | Age, gender, SES, education level, and car ownership (adjusted) | - Significant positive association was found between street density and reaching ≥ 30 min/day WT (OR= 1.71, 95%CI; 1.19-2.46), and CT (OR= 1.99, 95%CI; 1.24-3.19) |
| Chen et al, 2017 | Increasing the use of urban greenways in developing countries: A case study on Wutong Greenway in Shenzhen, China | Case study | Wutong Greenwa-y in Shenzhe-n, China | (N= 1257), [response rate; 96.7%], 53.8% males, 49.1% (15-34 yrs), 70.2% married, 37.2% had high school education, 50.0% owned house, and 45.1% had car | Random sampling of the green way users from three key access points | Self-reported questionnair-e (Leisure PA), Rating PA frequency on 5-point scale. | Self-reported questionnaire- Five variables;  Proximity to UGS entrances; (<500, 500-1000, 1000-3000, 3000-5000, or >5000m) | Age, gender, job status, marital status, education, and car ownership (no adjustment) | - Significant positive association was found between distances to the green way entrances and frequency of usage (<500m- OR= 1.9, 95%CI; 1.4–2.7; 500-1000m-OR= .8, 95%CI; 1.2–2.8; 1000–3000m-OR= 1.6, 95% CI; 1.0–2.4). |
| Cunningham-Myrie et al, 2015 | Associations between neighborhood effects and physical activity, obesity, and diabetes: The Jamaica Health and Lifestyle Survey 2008 | Cross-sectional | Jamaica | (N= 2,848), 36.9 ±0.1 yrs, 68.7% females | Multi-stage stratified random sampling | Self-reported questionnaire-locally developed (Leisure PA), using cut-off of 40 min/week | Observation & self-reported questionnaire- Three variables;  (a) Infrastructure to walk/ cycle, (b) Recreational areas existence, and (c) Recreational spaces proximity. | Age, gender, Fruit & vegetable consumption, enumeration district (ED), (adjusted for all covariates) | - Recreational areas existence (aOR=1.24, 95%CI; 1.04-1.48) showed significant unexpected positive association with achieving < 40 min/week PA. |
| Florindo et al, 2017 | Public open spaces and leisure-time walking in Brazilian adults | Cross-sectional | Sao Paulo, Southeastern Brazil | (N = 3145), 47.1± 18.8yrs, 57.7% women, 27.7% completed high school, 54.1% married | Multi-stage stratified random sampling | IPAQ-Long version: (Leisure-time walking), using cut-off of 150 min/week | GIS- (Arc Map version 10.3)- using radial buffers of 500, 1000, and 1500 m. Total of nine variables;  (a) Squares existence buffers, (b) Bike paths existence, and (c) Parks existence | Sex, age, education, marital status, body mass index, smoking, and residential region in Sao Paulo (north, mid-west, southeast, south, and east). (all adjusted) | - Only bike paths existence within 500-m buffers was associated with increased odds for achieving ≥150 min/week WL (aOR=1.53; 95%CI; 1.07–2.18). |
| Giehl et al,2016 | Built environment and walking behavior among Brazilian older adults: A population-based study | Cross-sectional | Florianó-polis, Brazil | (N= 1,705 older adults) [Response rate: 89.2%], 70.4 ± 8 yrs, 61.4% females, and 43.8% with ≤4 yrs of schooling | Multi-stage stratified random sampling | IPAQ- Long version (Walking for leisure & walking for transportation) using cut-off of 10 min/week | - GIS (ArcGIS 9.3)- Three variables; (a) Street density, (b) Street connectivity, and (c) Public open areas. - Census data- Total of four variables;  a) District income, (b) Population density, (c) Paved streets & (d) Sidewalks. | Age, gender, education (All adjusted) | - Significant positive association was found between street density- intermediate tertile classification- (aOR=1.47; 95%CI; 1.02–2.10), and district income-middle tertile classification (aOR=1.48; 95%CI; 1.04–2.12) and achieving ≥ 10 min/week walking for leisure. - Significant positive association was detected between high population density (aOR= 2.19; 95%CI; 1.40–3.42) & high street connectivity (aOR= 1.85; 95%CI; 1.16–2.94) and achieving ≥ 10 min/week walking for transpot. |
| Gómez et al, 2010a | Built environment attributes and walking patterns among the elderly population in Bogotá | Cross-sectional | Bogotá, Colombi-a | (N= 1,966 older adults) [response rate= 67.8%], 70.7±7.7 yrs, 63% females, 66% had primary eductaion level or less, and 35% had relatives living close/very close | Two-stage sampling design | IPAQ-short form (Combined walking for leisure and transport), using cut-off of 150 min/week | - GIS (ArcInfo, version 9) using a 500-m radius buffer- Total of four variables; a) Terrain slope, (b) Street connectivity, (c) Park density, and (d) Transport stations. - Self-reported questionnaire- Two variables; (a) Traffic safety, and (b) Sidewalks. | Gender, age groups, education level, limitation to engage in physical activity, slope, and SES (All adjusted) | - Significant positive association was found between park density- middle tertile (aOR: 1.42, 95%CI; 1.02–1.98) & perceived traffic safety (aOR: 1.50, 95%CI; 1.11–2.03) and  achieving ≥ 60 min/week total WT & WL. - Significant negative association was detected between land slope (aOR: 0.61, 95%CI; 0.38–0.97) & street connectivity-highest tertile- (aOR: 0.64, 95%CI; 0.44–0.93) and achieving ≥ 60 min/week total WT & WL. |
| Gomez et al, 2010b | Characteristics of the built environment associated with leisure-time physical activity among adults in Bogotá, Colombia: A multilevel study | Cross-sectional | Bogotá, Colombi-a | (N=1315) [response rate= 66%], 36± 13.5 yrs, 65% women, 24% had primary education or less | Stratified random sampling | IPAQ- Long version (Leisure time PA), using cut-off of 150 min/week | GIS (ArcInfo, version 9) - Six variables;  (a) Housing density, (b) Land-use mix, (c) Park density, (d) Transport stations proximity, (e) Bike paths availability, and (f) Land slope. | Gender, age group, education level, slope of the terrain and, environmental attributes finally included (all adjusted) | - Park density of (7.4- 25.2%) was positively associated with regular LTPA (aOR = 2.05, 95% CI = 1.13–3.72). |
| Gomes et al, 2011 | Walking for leisure among adults from three Brazilian cities and its association with perceived environment attributes and personal factors | Cross-sectional | Three capital states (Recife, Curitiba and Vitória), Brazil | (N=6,166), 45± 17 yrs, 58.8% females, 75.5% good health status, and 48.0% married. | Multi-stage stratified random sampling | IPAQ- Long version (walking for leisure), using cut-off of 150 min/week | NEWS-Brazil- Three variables;  (a) Sidewalks existence, (b) Traffic safety, and (c) Infrastructure to walk | Gender, Age categories, Education level, Marital status, Perceived health and BMI, (Adjusted) | - Significant positive association was found between lack of sidewalks and reaching ≥ 150 min/week walking for leisure (aOR= 1.5, 95%CI; 1.0-2.1). |
| Gul et al, 2018 | The effects of physical activity facilities on vigorous physical activity in T gated and non-gated neighborhoods | Cross-sectional | Karachi, Pakistan, 16 gated & non-gated neighbourhoods | (N=1200), 66.7% males, 44.1% (18-30yrs), 55.9% employed | Simple random sampling | IPAQ-SF; (Leisure time PA), using cut-off of 600 MET-min/week of VPA | Aerial map & observation methods- Total of three variables;  (a) < 3 PA facilities, (b) 3-6 PA facilities, and (c) >6 facilities | Gender, age, and employment status (NOT adjusted) | - A significant association was found between number of PAF and achieving ≥600 MET-min/week of VPA, R2= 37.4%, P value= 0.0001. |
| Hallal et al, 2010 | Association between perceived environmental attributes and physical activity among Adults in Recife, Brazil | Cross-sectional | Brazil, Recife (fifth largest Metropolitan) | (N= 2046), [response rate: 56%], 63% females, 37% aged 35–54 yrs, 58% had non-white skin color, 46% married, and 39% had a high school diploma. | Random-digit-dialing | IPAQ-Long version: (Leisure PA, transport PA & walking for leisure), using cut-off of 150 min/week | NEWS- Total of five variables;  (a)Sidewalks presence, (b) Access to nearby recreational places, (c) Total crime safety, (d) Traffic safety, and (e) Aesthetics. | Age, gender, skin colour, marital status, educational level (adjusted for all of them) | - A significant negative association was found between lack of sidewalks and achieving ≥150 min/week walking for recreation (aOR=0.5; 95%CI; 0.2–0.9). - Lack of sidewalks (aOR=0.6, 95%CI; 0.3–0.9) and reduced access to nearby recreational amenities (aOR=0.7, 95%CI; 0.5–1.0) were negatively associated with achieving ≥150 min/week leisure time MVPA |
| Hino et al, 2011 | The built environment and recreational physical activity among adults in Curitiba, Brazil | Cross-sectional | Curitiba, Brazil | (N=1206), [response rate=93.2%], 62.3% women, 42.3% aged (35-54), 38.1% had high school education, 60.0% married, 71.7% had car, and 56.1% with normal BMI | Stratified and random sampling | IPAQ-LF; (WL & LTPA), using cut-off of 150 min/week | GIS (ArcGIS 9.2)- Total of three variables; (a) Recreational amenities availability, (b) Recreational facilities (sport center) proximity, and (c) District income. | Sex, age, BMI, education, marital status, and car ownership (adjusted) | - Significant positive associations between recreational facilities density- (aOR=1.89; 95%CI; 1.21–2.97) & recreational facilities proximity- middle tertile (aOR=2.26; 95%CI; 1.04–2.49) and achieving ≥150 min/week WL. - Significant positive association between recreational facilities density- (aOR=1.52; 95%CI; 1.11–2.09) and achieving ≥150 min/week LTPA**.** |
| Hino et al, 2014 | Built environment and physical activity for transportation in adults from Curitiba, Brazil | Cross-sectional | Curitiba, Brazil | (N= 1,206) 62.3% Females, 42.3% (35-45yrs), 60% married, 71.7% own car, and 56.1% normal BMI/underweight | Random-digit-dialing | IPAQ-Long version: (WT and CT), using cut-off of 150 min/week | GIS using a 500-m radius buffer- Nine variables;   (a) Traffic safety, (b) Residential density, (c) Commercial density, (d) Street density, (e) Connectivity (f) Land slope, (g) Bike path density, (h) Bus stop proximity, and (i) Bike path proximity. | Sex, age, BMI, educational, marital status, and car ownership. (all adjusted) | - Significant positive associations between residential density- middle tertile (aOR=1.25; 95%CI; 1.02–1.53) & commercial density- middle tertile (aOR=1.47; 95%CI; 1.13–1.91) and achieving ≥10 min/week WT. - Significant negative associations between district income, highest tertile (aOR=0.26; 95%CI; 0.08–0.81), traffic safety (aOR=0.27; 95%CI; 0.09–0.84), and residential density, middle tertile (aOR=0.53; 95%CI; 0.34–0.83), and achieving ≥10 min/week CT. |
| Jaime et al, 2011 | Investigating environmental determinants of diet, physical activity, and overweight among adults in Sao Paulo, Brazil | Cross-sectional | Sao Paulo, Brazil, 31 submuni-cipalities | (N=2,122 adults aged ≥18yrs) | Simple random sampling | Self-reported PA questionnaire (LTPA); using cut-off of 90 min/week MVPA | GIS & census data- Three variables; (a) Park density, (b) Transport system accessibility, and (c) Crime safety | SES of areas (adjusted) | - Significant negative correlation between crime safety (feeling unsafe) and achieving ≥90 min/week MVPA (r= -0.395). |
| Jáuregui et al, 2016 | Perceived neighborhood environment and physical activity: The International Physical Activity and Environment Network Adult Study in Mexico | Cross-sectional | Cuernavaca, Mexico | (N=659), 42yrs (95% CI=40.7, 43.2), 50.9% females, 64.6% married, and 53.4% owned car | Multi-stage stratified random sampling | Accelerometer (GT3X Actigraph)- wearing the device for 7 days, (any/all PA), using cutoff of 150 min/week MVPA | NEWS- Six variables;  (a)Aesthetics, (b) Pedestrian infrastructure/sidewalks, (c) Traffic safety, (d) Crime safety, (e) Parks proximity, and (f) Transit stops proximity. | Sex, age, marital status, SES, motor vehicle ownership, educational attainment, and accelerometer wear time (adjusted) | - Significant positive association was found between perceived crime safety-among males- (aOR=1.29, 95%CI; 1.07- 1.57) & parks proximity (aOR= 1.12, 95%CI; 1.01- 1.24) and reaching ≥ 150 min/week MVPA. |
| Jáuregui et al, 2017 | Perceived neighborhood environmental attributes associated with leisure-time and transport physical activity in Mexican adults | Cross-sectional | Cuernavaca, Mexico. | (N= 668 Mexican adults), 42.0 yrs (95% CI: 40.7–43.2), 51.2% women, and 53.4% owned a car | Multi-stage stratified random sampling | IPAQ-Long version: (leisure time PA, walking for leisure & transport-related PA); using cut-off of 150 min/week | NEWS- Total of seven variables;  (a)Aesthetics, (b) Land-use mix diversity, (c) Safety to walk/cycle, (d) Traffic safety, (e) Crime safety, (f) Proximity to parks, and (g)Transit stops access/proximity. | Sex, age, marital status, SES, motor vehicle ownership, and educational attainment (adjusted) | - Perceived aesthetics was positively associated with leisure time MVPA participation [exp (b)= 1.33, exp 95%CI; 1.04-1.70], and duration [exp (b)= 1.67, exp 95%CI; 1.12-2.49]. - Significant positive association was found between traffic safety and walking for leisure duration [exp (b)= 1.33, exp 95%CI; 1.18-3.13]. |
| Jia et al, 2014 | The association between walking and perceived environment in Chinese community residents: A cross- sectional study | Cross-sectional | Minhang district of Shanghai, China. | (N= 1528), [response rate; 84.9%], (15-75 yrs), 50.8% female, 74.6% married, 32.7% had a college/university degree, and 45.3% employed. | Multi-stage stratified random sampling | IPAQ-long version (walking for leisure and walking for transport)- using cut-off of 90 min/week | NEWS- Total of four variables;  (a) Services accessibility, (b)Aesthetics , (c) Traffic safety, and (d) Crime safety. | Gender, age, education levels, employment status, body height and weight, and marital status (adjusted) | - Significant positive association was found between service accessibility and reaching > 90 min/week WL (aOR =1.062, 95%CI; 1.016-1.110). - Significant positive association was detected between service accessibility and reaching > 90 min/week WT (aOR =1.053, 95%CI; 1.008-1.100). |
| Katulanda et al, 2012 | Physical activity patterns and correlates among adults from a developing country: The Sri Lanka Diabetes and Cardiovascular Study | Cross-sectional | Sri Lanka | (N=4485), [response rate; 89.7 %], 39.5 % males, 46.1± 15.1 yrs, 52.6 % unemployed, and 86.4 % Sinhalese ethnicity. | Simple random sampling | IPAQ-SF; (All PA), using cut-off of 150 min/week MVPA | Census data (urban vs rural settings) | Age, sex, job status, ethnicity (not adjusted) | - Urbanicity is significantly positively associated with achieving < 150 min/week MVPA (OR=2.5, 95%CI; 2.1–2.9). |
| Koyanag-i et al, 2018 | Correlates of low physical activity across 46 low- and middle-income countries: A cross-sectional analysis of community-based data | Cross-sectional | 46 countries (Africa 19, Americas 6, Asia 13, Europe 8), which were all LMICs (21 low-, and 25 middle-income countries) | (N= 206,356) [response rate; 98.5%] , 38.4± 16.0 yrs, 66.3% married, 49.2% males, 33.7% completed secondary education, and 42.5% unemployed. | Multi-stage stratified random sampling | IPAQ- (total PA), using cut-off of 150 min/week MVPA | Self-reported questionnaire, Urban vs Rural areas | Sex, age, marital status, education, employment status (adjusted) | - Urbanicity is significantly positively associated with achieving < 150 min/week MVPA (OR=1.56, 95%CI; 1.43–1.71). |
| Malamb-o et al, 2018 | The relationship between objectively- measured attributes of the built environment and selected cardiovascular risk factors in a South African urban setting | Cross-sectional | Cape Town, South Africa | (N=341), 56.1 ± 10.6yrs, 77.4% females, 41.9% unmarried, 61.9% completed secondary school, 61.9% unemployed, and 92.7% did not own a car | N/A | Accelerometer (ActiGraph GT3X), using cut-off of ≥9643 counts/min | GIS (ArcGIS 9.3 ESRI software), using radial buffers of 500, 1000, and 1600 m- Total of two variables;  (a) Public services proximity [police station, health centers, open areas], and (b) Transit stops proximity | Age, gender, marital status, education level, employment status, family income, smoking, alcohol use and ownership of motor cars (adjusted age, sex) | - Only community centers proximity within 1600-m buffers was significantly negatively associated with achieving ≥9643 counts/min (β= -69.30, 95%CI; − 134.92 to − 3.70). |
| Oyeyemi et al, 2011 | Perceived environmental correlates of physical activity and walking in African young adults | Cross-sectional | Ibada, Oyo State in Southwestern Nigeria. | (N= 1006 students from The University of Ibadan), [response rate; 85.8%] 22± 2.78 yrs, 50.7% females, and BMI 22.08± 3.95 kg/m2 | Multi-stage stratified random sampling | IPAQ-SF- (combined WL & WT and all PA types), using cut-off of 150 min/week | PANES- 11 variables;  (a) Residential density, (b) Shops accessibility, (c) Transit stop accessibility, (d) Recreational amenities, (e) Bike path availability, (f) Sidewalks, (g) Crime safety at night, (h) Crime safety during the day, (i) Traffic safety, (k) Aesthetics, and (l) Street connectivity. | Age, BMI, and gender (adjusted) | - Significant positive association was found between perceived safety from crime at night (aOR=1.53, 95%CI; 1.02-2.35) & perceived pleasant aesthetics (aOR=1.90, 95%CI; 1.33-2.69) and reaching ≥ 150 min/week total WL & WT. - Significant positive association was detected between perceived transit stop accessibility and reaching ≥ 150 min/week MPA (excluding walking) (aOR= 1.41, CI; 1.05–1.90). |
| Oyeyemi et al, 2012 | Perceived crime and traffic safety is related to physical activity among adults in Nigeria | Cross-sectional | Maiduguri, North Eastern Nigeria | (N=219), 34.9 ± 8.8 yrs, 60.7% males, 72.6% married, 72.2% employed, 49% had > secondary education | Multi-stage stratified random sampling | 1) IPAQ-SF, and 2) accelerometer, (combined WL & WT and all PA types), using cut-off of 150 min/week | PANES- Total of three variables;  (a) Traffic safety, (b) Crime safety during day, and (c) Crime safety at night. | Age, gender, neighborhood location, employment status, and educational level (adjusted) | - Significant positive association was noted between perceived crime safety during the day (aOR= 5.92, CI; 1.38–60.59) & at night (aOR= 6.99, CI; 2.71–18.04) and reaching ≥ 150 min/week total WL & WT. - Significant positive association was found between perceived crime safety at night (aOR=1.68, 95%CI; 1.07–3.64) and achieving ≥ 150 min/week MVPA |
| Parra et al, 2011 | Perceived environmental correlates of physical activity for leisure and transportation in Curitiba, Brazil | Cross-sectional | Curitiba, Brazil. | (N = 2,097), 63% females, 42% (35–54 years), 66% had less than high school, and 71% had ≥ one car. | Stratified and random sampling | IPAQ-LF; (LTPA, WL, WT, CT, and total PA), using cut-off of 150 min/week. | NEWS- Total of four variables; (a) Proximity to services, (b) Traffic safety, (c) Crime safety, and (d) Accessibility (sidewalks, land slope, and bike path availability). | Age, gender, education level, and car ownership (adjusted) | - Significant positive association was found between crime safety (aOR: 1.5, 95%CI; 1.0–2.1) and WT. - Significant positive association was found between proximity to services (aOR: 2.5, 95%CI; 1.2–5.2) and CT. - Significant positive association was found between accessibility (aOR: 1.7, 95%CI; 1.2–2.4) and MVPA for leisure. |
| Reis et al, 2013a | Bicycling and Walking for Transportation in Three Brazilian Cities | Cross-sectional | Curitiba, Vitoria, and Recife in Brazil | (N=6,166), 45± 17 yrs, 58.8% females, 39.2% with high school education, and 48.0% married. | Multi-stage stratified random sampling | IPAQ-LF; (WT & CT), using cut-off of 150 min/week for WT | NEWS- Total of 4 variables; (a) Sidewalks, (b) Traffic safety, (c) Safety to walk/cycle during day, and (d) Safety to walk/cycle at night | Gender, Age categories, Education level, Marital status, Perceived health and BMI, (Adjusted) | - No significant associations were found between any BE variable and WT or CT. |
| Reis et al, 2013b | Walkability and physical activity: Findings from Curitiba, Brazil | Cross-sectional | Curitiba, Southern Brazil, 32 census tracts varied in income and walkabili-ty | (N=697), [Response rate; 66.4%], (18-65 yrs), 52.94% females, 57.82% married, 45.77% had one car, and 51.79% had children at home. | Simple random sampling | IPAQ- Long version (walking for leisure, walking for transportation, leisure MVPA), using cut-off of 150 min/week | GIS & census data- Total of two variables; (a) walkability index [estimated utilizing three indicators, land-use mix, housing density, connectivity], and (b) District income | Area income, gender, age, education, marital status, number of children at living at home, car ownership, and time living in the neighborhood (adjusted) | - An independent positive association was noted between walkability and achieving ≥150 min/week WL (aOR=2.10, 95%CI; 1.31- 3.37). - A positive association was found between walkability and leisure- time MVPA (aOR=1.57, 95%CI;1.06-2.32). |
| Rech et al, 2012 | Neighborhood safety and physical inactivity in adults from Curitiba, Brazil | Cross-sectional | Curitiba, Brazil. | (N=1261 adults aged 18–69), 62% females, 51.4% had normal weight, 50.0% with medium SES, 57.5% married, and 72.2% had children, | Stratified and random sampling | IPAQ-LF; (WL, WT & LTPA), reported weekly frequency and time spent in each domain | NEWS- Total of 2 variables; (a) Crime safety during the day, and (b) Crime safety at night | Sex, age, nutritional status, SES, marital status, children, and private transport use (adjusted) | - Only significant negative association was noted between feeling unsafe from crime at night and WT (aPR: 0.73, 95%CI; 0.57–0.94) |
| Rech et al, 2014 | Personal, social and environmental correlates of physical activity in adults from Curitiba, Brazil | Cross-sectional | Curitiba, Brazil | (N=1,461 adults), 46.1% aged (40-59.9), 63.7% women, 49.3% medium SES, 57.4% married, and 51.6% with normal weight | Stratified and random sampling | IPAQ-LF; (WL & LTPA), using cut-off of 150 min/week | NEWS- Total of 4 variables; (a) Residential density, (b) leisure facilities proximity, (c) aesthetics, and (d) crime safety | Age, gender, marital status, SES, and BMI (adjusted) | - Significant positive associations were found between neighbourhood aesthetics (aOR=1.63; 95%CI; 1.20–2.21), and leisure facilties proximity (aOR=1.35; 95%CI; 1.02–1.80) and achieving ≥150 min/week WL. - Significant positive associations were noted between neighbourhood aesthetics (aOR=1.36; 95%CI; 1.01–1.84), and feeling safe from crime (aOR=1.36; 95%CI; 1.02–1.84), and achieving ≥150 min/week MVPA for leisure |
| Trude et al, 2016 | Factors associated with physical inactivity and sedentary behavior among women living in the urban area of Santos City, Brazil | Cross-sectional | Santos, Brazil | (N=538 women), 66.5% aged >30yrs, 56.9% employed, 58.0% had > one child, 55.6% had highest SES, and 53.9% owned a car | Stratified and random sampling | IPAQ-short form (total PA), using cut-off of 150 min/week | Self-reported questionnaire- Total of two variables;  (a) Safety to cycle, and (b) Recreational amenities proximity. | Women’s age, socioeconomic level, women’s daily working hours, women’s child age (all adjusted) | - Significant positive unexpected association was noted between perceived safety for cycling and achieving < 150 min/week MVPA (aOR=1.50, 95%CI; 1.05–2.16). |
| Vancam-pfort et al, 2019 | Associations of the built environment with physical activity and sedentary time in Ugandan outpatients with mental health problems | Cross-sectional | Uganda, Kampala | (N=99 patients with mental health problems), 31.1± 8.6 yrs, BMI 23.2± 4.0 kg/m2, 78.8% males, 24.2% married, and 35.3% with low education level. | Outpatie-nts invited from hospital | SIMPAQ3 (Total walking for transport & leisure, and leisure-time PA excluding walking) | NEWS- Eight variables; (a) Services proximity, (b) Recreation amenities existence, (c) Sidewalks, (d) Bike path availability, (e) Aesthetics, (f) Traffic safety, (g) Crime safety, and (h) Safety to walk/cycle. | Age, gender, marital status, education, BMI, mental disorders (adjusted) | - 8% of the variance in time spent on PA was elaborated by the availability of recreational amenities. - 13% of the variance in time spent on total WT and WL was elaborated by services accessibility and the availability of sidewalks. |

PA= physical activity; BE= built environment; IPAQ-LF= International Physical Activity Questionnaire- long form; NEWS= Neighbourhood Environment Walkability Scale; SIMPAQ= Simple Physical Activity Questionnaire; PANES= Physical Activity Neighbourhood Environment Scale; BMI= body mass index; WL= walking for leisure; WT= walking for transportation; CT= cycling for transportation; LTPA= leisure-time physical activity; TRPA= transport-related physical activity; MVPA= moderate to vigorous physical activity, UGS= urban green spaces; SES= socioeconomic status; GIS= geographic information system
