# Supplementary material for: Built environment correlates of physical activity in low- and middle-income countries: A systematic review
Source: PLoS One. 2020 Mar 17;15(3):e0230454. doi: 10.1371/journal.pone.0230454 (PMC7077823; doi:10.1371/journal.pone.0230454)
Supplement: S4 Appendix — (DOCX) [file pone.0230454.s004.docx]

**S4 Appendix. Classification of data according to PA domains- Leisure-time physical activity**

| **Leisure-time PA** | | **Built environment** | | **Analysis** | **Main findings** | **Reference** |
| --- | --- | --- | --- | --- | --- | --- |
| **PA tool** | **PA variables** | **BE tool** | **BE variables** |  |  |  |
| IPAQ-LF | - ≥150 min/week MVPA - < 150 min/week MVPA | NEWS-India | *Total of eight variables*  **(a)** Residential density, **(b)** Land use mix-diversity, **(c)** Land use mix-access, **(d)** Street connectivity, **(e)** Infrastructure and safety to walk/cycle, **(f)** Aesthetics, (**g)** Traffic safety, and (**h)** Crimes safety. | Logistic analysis examining association between NEWS subscale scores & achieving ≥150 min/week MVPA | Significant negative association between residential density and achieving ≥150 min/week MVPA (aOR=0.6, 95%CI; 0.4-1.0). | [8] |
| Tailored, self-reported PA question-naire | - Rating PA frequency in UGS on 5-point scale. - Rating PA duration in UGS on 5-point scale (<15 min, 15–30 min, 30 min to 1 h, 1–2 h, and >2 h) | Tailored, self-reported BE/UGS attributes questionnaire | *Total of three variables*  **(a)**Distance to the nearest UGS, **(b)** Aesthetics, and **(c)** Recreational facilities existence. | Multivariate analysis examining association between UGS attributes and PA frequency/ duration | - Significant positive association between PA frequency in UGS and many trees existence (b=0.326, 95% CI; 0.105–546) and near distance to UGS (b=0.097, 95% CI; 0.004–0.197). - Exercise equipment (b=0.147, 95%CI; -0.003–0.297), and picnic places (b=0.223, 95%CI; 0.083–0.363) were positively associated with PA duration in UGS. | [39] |
| IPAQ-LF | - ≥150 min/week MVPA - < 150 min/week MVPA | NEWS- Brazil | *Total of five variables*  **(a)** Sidewalks existence, (**b)** Green areas existence, (**c)** Traffic safety, (**d)** Cross-walks existence, and **(e)** Crime existence. | Poisson multivariable analysis examining association between NEWS subscale scores & achieving < 150 min/week MVPA | - Significant positive association between existence of crimes (aPR= 1.10; 95%CI; 1.00–1.22) and achieving < 150 min/week MVPA. - Significant negative association between existence of green areas (aPR=0.87, 95%CI; 0.81–0.94) and achieving < 150 min/week MVPA. | [44] |
| Self-reported-PA question-naire | - Rating PA frequency in UGS on 5-point scale. | Self-reported questionnaire | *Total of five variables*  Proximity to UGS entrances; (<500, 500-1000, 1000-3000, 3000-5000, or >5000m) | Logistic analysis examining association between proximity to UGS entrances and a) PA frequency b) PA duration | Significant positive association between distances to UGS entrances and frequency of UGS usage (<500m- OR= 1.9, 95%CI; 1.4–2.7; 500-1000m-OR= 1.8, 95%CI; 1.2–2.8; 1000–3000m-OR= 1.6, 95% CI; 1.0–2.4). | [45] |
| Tailored, self-reported PA questionnaire | - >40 min/week PA - <40 min/week PA | - Observation - self-reported BE safety perception questionnaire | *Total of three variables*  **(a)** Safety to walk/cycle, (**b)** Recreational areas existence, and **(c)** Recreational spaces proximity. | Logistic analysis examining association between BE attributes & achieving < 40 min/week PA. | Recreational areas existence (aOR=1.24, 95%CI; 1.04-1.48) showed significant unexpected positive association with achieving < 40 min/week PA. | [42] |
| IPAQ-LF | - Regular leisure PA (≥150 min/week) - Irregular leisure PA (10-149 min/week) - Inactivity (<10 min/week) | GIS (ArcInfo, version 9) | *Total of four variables*  (**a)** Park density, (**b)** Transportation system stations proximity, (**c)** Bike paths availability, and **(d)** Land slope. | Logistic analysis examining association between BE attributes & achieving ≥ 150 min/week leisure PA. | - Park density of (7.4- 25.2%) was positively associated with regular leisure PA (aOR = 2.05, 95% CI = 1.13–3.72). - Terrain slope of ≥4% was negatively associated with regular leisure PA (aOR= 0.37, 95%CI; 0.14–0.97). | [19] |
| IPAQ-SF | - ≥600 MET-min/week of VPA - <600 MET-min/week of VPA | - Aerial maps - Observation | *Total of three variables*  (**a)** < 3 PAF, **(b)** 3-6 PAF, and **(c)** >6 PAF in a neighbourhood. | Gamma test examining association between number of PAF in a neighbourhood & achieving ≥600 MET-min/week of VPA | A significant association between no. of PAF and achieving ≥600 MET-min/week of VPA, R^2^= 37.4%, P value= 0.0001. | [40] |
| IPAQ-LF | - ≥150 min/week MVPA - < 150 min/week MVPA | NEWS- Brazil | *Total of five variables*  **(a)**Sidewalks presence**, (b)** Access to nearby recreational places, **(c)** Total crime safety, **(d)** Traffic safety, and **(e)** Aesthetics. | Logistic analysis examining association between NEWS scores & achieving ≥150 min/week MVPA. | Lack of sidewalks (aOR=0.6, 95%CI; 0.3–0.9) and reduced access to nearby recreational amenities (aOR=0.7, 95%CI; 0.5–1.0) were negatively associated with achieving ≥150 min/week MVPA. | [46] |
| IPAQ-LF | - ≥150 min/week MVPA - < 150 min/week MVPA | GIS (ArcGIS 9.2) | *Total of three variables*  **(a)** Recreational amenities (gym facilities) availability/density, **(b)** Recreational facilities (sport center) proximity, and **(c)** District income. | Logistic analysis examining association between BE variables & achieving ≥150 min/week MVPA. | - Significant positive associations between recreational facilities density- (aOR=1.52; 95%CI; 1.11–2.09) and district income-highest tertile (aOR= 2.97; 95%CI; 1.50–5.89) & achieving ≥150 min/week leisure PA. | [43] |
| Self-reported PA question-naire | - ≥90 min/week MVPA - <90 min/week MVPA | - GIS - Census data | *Total of three variables*  **(a)** Park density, **(b)** Transport system accessibility, and **(c)** Crime safety | Correlation analysis examining association between BE variables & achieving ≥90 min/week MVPA | Significant negative correlation between crime safety (feeling unsafe), and achieving ≥90 min/week MVPA (r= -0.395). | [38] |
| IPAQ-LF | - ≥150 min/week MVPA - < 150 min/week MVPA | NEWS- America | *Total of seven variables*  **(a)**Aesthetics, **(b)** Land-use mix diversity, **(c)** Safety to walk/cycle, **(d)** Traffic safety, **(e)** Crime safety, **(f)** Proximity to parks, and **(g)**Transit stops access/proximity. | Hurdle regression models examining association between NEWS scores and **a)** PA participation, and **b)** PA duration. | Perceived aesthetics was positively associated with MVPA participation [exp (b)= 1.33, exp 95%CI; 1.04-1.70], and duration [exp (b)= 1.67, exp 95%CI; 1.12-2.49]. | [36] |
| IPAQ-LF | PA continuous scores- (weekly frequency* PA daily duration in min) | NEWS- Brazil | *NEWS- Total of four variables*  **(a)** Proximity to services, **(b)** Traffic safety, **(c)** Crime safety, and **(d)** Accessibility (sidewalks, land slope, and bike path availability). | Logistic analysis examining association between each BE variable and leisure PA scores | Significant positive association was found between accessibility (aOR: 1.7, 95%CI; 1.2–2.4) and MVPA for leisure. | [33] |
| IPAQ-LF | Self-reported frequency and time spent in each domain | NEWS- Brazil | *Total of two variables*  (a) Crime safety during the day, and (b) Crime safety at night | Poisson regression analysis examining associations between BE variables and leisure PA | No significant associations were found between any BE variable and leisure PA. | [48] |
| IPAQ-LF | - ≥150 min/week MVPA - < 150 min/week MVPA | NEWS- Brazil | *Total of four variables;*  **(a)** Residential density, **(b)** Leisure facilities proximity, **(c)** Aesthetics, and **(d)** Crime safety | Logistic analysis examining association between each BE variable and achieving ≥150 min/week MVPA | Significant positive associations were noted between neighbourhood aesthetics (aOR=1.36; 95%CI; 1.01–1.84), and feeling safe from crime (aOR=1.36; 95%CI; 1.02–1.84), and achieving ≥150 min/week MVPA | [37] |
| IPAQ-LF | - ≥150 min/week MVPA - < 150 min/week MVPA | - GIS - Census data | *Total of two variables*  **(a)** walkability index [estimated utilizing three indicators, land-use mix, housing density, connectivity], and (**b)** District income | Logistic analysis examining association between each BE variable and achieving ≥150 min/week MVPA | - An independent positive association between walkability and leisure- time MVPA (aOR=1.57, 95%CI;1.06-2.32). - A significant positive association between district income and leisure-time MVPA (aOR=1.70, 95% CI;1.06-2.74). | [47] |
| SIMPAQ | Time spent on leisure PA in min/day (excluding walking). | NEWS-Africa | *Total of eight variables*  **(a)** Services proximity, **(b)** Recreation amenities existence, **(c)** Sidewalks, **(d)** Bike path availability, **(e)** Aesthetics, **(f)** Traffic safety, **(g)** Crime safety, and **(h)** Safety to walk/cycle. | Multivariate analysis examining association between NEWS subscale scores & time of leisure PA (excluding walking). | 8% of the variance in time spent on PA was elaborated by the availability of recreational amenities. | [41] |

PA= physical activity; BE= built environment; IPAQ-LF= International Physical Activity Questionnaire- long form; NEWS= Neighbourhood Environment Walkability Scale; SIMPAQ= Simple Physical Activity Questionnaire; PANES= Physical Activity Neighbourhood Environment Scale; BMI= body mass index; MVPA= moderate to vigorous physical activity, UGS= urban green spaces; SES= socioeconomic status; GIS= geographic information system

**S4 Appendix. Classification of data according to PA domains- Transport-related physical activity**

| **Transport-related PA** | | **Built environment** | | **Analysis** | **Main findings** | **Reference** |
| --- | --- | --- | --- | --- | --- | --- |
| **PA tool** | **PA variables** | **BE tool** | **BE variables** |  |  |  |
| IPAQ-LF | - ≥150 min/week MVPA - < 150 min/week MVPA | NEWS-India | *Total of eight variables*  **(a)** Residential density, **(b)** Land use mix-diversity, **(c)** Land use mix-access, **(d)** Street connectivity, **(e)** Infrastructure and safety to walk/cycle, **(f)** Aesthetics, (**g)** Traffic safety, and (**h)** Crimes safety. | Logistic analysis examining association between NEWS scores and achieving ≥150 min/week MVPA | - Significant positive association between residential density (aOR= 1.9, 95%CI; 1.2-3.2) and land use mix-diversity (aOR= 2.1, 95%CI; 1.2-3.6) and achieving ≥150 min/week MVPA. - Significant negative association between street connectivity (aOR=0.6, 95%CI; 0.3-1.0), aesthetics (aOR= 0.4, 95%CI; 0.2-0.9), and crime safety (aOR= 0.5, 95%CI; 0.3-0.9), and achieving ≥150 min/week MVPA. | [8] |
| Tailored, self-reported PA questionnaire | - Active commuting (walking/cycling) - Inactive commuting (public/private transport) - Multi-modal (walk/bicycle & transport modes) | NEWS- India | *Total of nine variables*  **(a)** Residential density, (**b)** Land use mix-diversity, (**c)** Land use mix-access, (**d)** Street connectivity, (**e)** Infrastructure and safety to walk/cycle, (**f)** Aesthetics, (**g)** Traffic safety, and (**h)** Crime safety, and **(i)** Transit stop access/proximity | Logistic analysis examining association between NEWS scores and active or multi-modal commuting. | - A significant positive association between transit stop proximity (aOR =5.0, 95%CI; 1.7–14.4) and land-use mix diversity (aOR =6.8, 95%CI; 2.3–20.6) and active/multimodal commuting. - A significant negative association between aesthetics (aOR =0.2, 95%CI; 0.0–1.0), crime safety (aOR =0.2, 95%CI; 0.1–0.6), and street connectivity (aOR =0.2, 95%CI; 0.1–0.6), and likelihood of active/multimodal commuting. | [49] |
| IPAQ-LF | - ≥150 min/week MVPA - < 150 min/week MVPA | NEWS- Brazil | *Total of five variables*  **(a)** Sidewalks existence, (**b)** Green areas existence, (**c)** Traffic safety to walk/cycle, (**d)** Cross-walks existence, and (**e)** Crime existence, | Poisson multivariable analysis examining association between NEWS subscale scores & achieving < 150 min/week MVPA | - Significant positive association between difficulty to walk/cycle (traffic unsafety) and achieving < 150 min/week transport-related MVPA (aPR=1.18, 95%CI; 1.04–1.33). | [44] |
| IPAQ-LF | - ≥150 min/week MVPA - < 150 min/week MVPA | NEWS-A | *Total of five variables*  **(a)**Sidewalks presence**, (b)** Access to nearby recreational places, **(c)** Total crime safety, **(d)** Traffic safety, and **(e)** Aesthetics. | Logistic analysis examining association between NEWS scores & achieving ≥150 min/week MVPA | Significant positive association between unpleasant aesthetics and achieving ≥ 150 min/week transport-related MVPA (aOR=1.5, 95%CI; 1.2–2.0) {unpredicted}. | [46] |
| IPAQ-LF | - ≥150 min/week MVPA - < 150 min/week MVPA | NEWS- America | *Total of seven variables*  **(a)**Aesthetics, **(b)** Land-use mix diversity, **(c)** Safety to walk/cycle, **(d)** Traffic safety, **(e)** Crime safety, **(f)** Proximity to parks, and **(g)**Transit stops access/proximity. | Hurdle regression models examining association between NEWS scores and **a)** PA participation, and **b)** PA duration. | - A significant negative association between parks proximity and PA participation (exp[B] = 0.78, 95%CI = 0.63–0.97), and duration (exp[B] = 0.69, 95%CI = 0.54–0.88). | [36] |

PA= physical activity; BE= built environment; IPAQ-LF= International Physical Activity Questionnaire- long form; NEWS= Neighbourhood Environment Walkability Scale; SIMPAQ= Simple Physical Activity Questionnaire; PANES= Physical Activity Neighbourhood Environment Scale; BMI= body mass index; MVPA= moderate to vigorous physical activity, UGS= urban green spaces; SES= socioeconomic status; GIS= geographic information system

**S4 Appendix. Classification of data according to PA domains- Transport cycling & leisure and/or transport walking**

| **Transport cycling & leisure and/or transport walking** | | **Built environment** | | **Analysis** | **Main findings** | **Reference** |
| --- | --- | --- | --- | --- | --- | --- |
| **PA tool, type** | **PA variables** | **BE tool** | **BE variables** |  |  |  |
| IPAQ, transport walking or cycling | - Walking ≥30 min/day - Walking <30 min/day | GIS- using 500-m buffer-rings and 1000-m peripheral buffers | - Street density ≥ 0.20 - Street density < 0.20 | Hierarchical nonlinear model predicting relationships between BE variables and transport walking or cycling for ≥30 min/day | Significant positive association was found between street density and reaching ≥ 30 min/day transport walking (OR= 1.71, 95%CI; 1.19-2.46) | [35] |
|  | - Cycling ≥30 min/day - Cycling <30 min/day |  |  |  | Significant positive association was found between street density and reaching ≥ 30 min/day transport cycling (OR= 1.99, 95%CI; 1.24-3.19) |  |
| IPAQ-LF, leisure walking | - Walking ≥150 min/week - Walking <150 min/week | GIS- (Arc Map 10.3) | *Total of nine variables*  **(a)** Squares existence within 500, 1000 & 1500m buffers, **(b)** Bike paths existence within 500, 1000 & 1500 m buffers, and **(c)** Parks existence within 500, 1000 & 1500 m buffers. | Logistic analysis examining association between each BE variable and achieving ≥150 min/week leisure walking | Only bike paths existence within 500-m buffers was associated with increased odds for achieving ≥150 min/week leisure walking (aOR=1.53; 95%CI; 1.07–2.18). | [14] |
| IPAQ-LF, leisure walking | - Walking ≥10 min/week - Walking <10 min/week | - GIS (ArcGIS 9.3) - Census data | *Total of three variables (from GIS)*  **(a)** Street density, **(b)** Street connectivity, and **(c)** Public open areas  *Total of four variables (from census)*  **a)** District income, **(b)** Population density, **(c)** Proportion of paved streets, and **(d)** Proportion of sidewalks. | Logistic analysis examining association between each BE variable and achieving ≥ 10 min/week leisure walking | Significant positive association between street density- intermediate tertile classification- (aOR=1.47; 95%CI; 1.02–2.10), and district income-middle tertile classification (aOR=1.48; 95%CI; 1.04–2.12) and achieving ≥ 10 min/week leisure walking | [50] |
| IPAQ-LF, transport walking |  |  |  | Logistic analysis examining association between each BE variable & achieving ≥ 10 min/week transport walking | - Significant positive association between high population density (aOR= 2.19; 95%CI; 1.40–3.42) & high street connectivity (aOR= 1.85; 95%CI; 1.16–2.94) and achieving ≥ 10 min/week transport walking. - Significant positive association between high sidewalks percentage (OR: 1.77, 95%CI; 1.11–2.83) & paved streets (middle tertile-aOR: 1.61, 95%CI; 1.04–2.49; highest tertile- aOR: 2.1, 95%CI; 1.36–3.27) and achieving ≥ 10 min/week transport walking. |  |
| IPAQ-SF, combined leisure & transport walking | - Walking ≥ 60 min/week - Walking ≥150 min/week | - GIS (ArcInfo, version 9), using 500-m radius buffer - Tailored, self-reported BE attributes questionnaire | *Total of four variables (from GIS)*  **a)** Terrain slope, **(b)** Street connectivity, **(c)** Park density, and **(d)** Transport stations.  *Total of two variables (from questionnaire/environmental perception questions)*  **a)** Traffic safety, and **(b)** Sidewalks. | Multivariate analysis examining association between BE attributes & achieving ≥ 60 min/week & ≥150 min/week. | - Significant positive association between park density- middle tertile (aOR: 1.42, 95%CI; 1.02–1.98) & perceived traffic safety (aOR: 1.50, 95%CI; 1.11–2.03) and   achieving ≥ 60 min/week total transport walking & leisure walking.   - Significant negative association between land slope (aOR: 0.61, 95%CI; 0.38–0.97) & street connectivity-highest tertile- (aOR: 0.64, 95%CI; 0.44–0.93) and achieving ≥ 60 min/week total transport walking & leisure walking. | [22] |
| IPAQ-LF leisure walking | - Walking ≥150 min/week - Walking <150 min/week | - NEWS- Brazil | *Total of three variables*  **(a)** Sidewalks existence, **(b)** Traffic safety, and **(c)** Safety to walk | Logistic analysis examining association between NEWS scores & reaching ≥ 150 min/week leisure walking. | Significant positive association between lack of sidewalks and reaching ≥ 150 min/week leisure walking (aOR= 1.5, 95%CI; 1.0-2.1). | [20] |
| IPAQ-LF leisure walking | - Walking ≥150 min/week - Walking <150 min/week | NEWS-A | *Total of five variables*  **(a)**Sidewalks presence**, (b)** Access to nearby recreational places, **(c)** Total crime safety, **(d)** Traffic safety, and **(e)** Aesthetics. | Logistic analysis examining association between NEWS scores and achieving ≥150 min/week leisure walking | A significant negative association between lack of sidewalks and achieving ≥150 min/week leisure walking (aOR=0.5; 95%CI; 0.2–0.9). | [46] |
| IPAQ-LF leisure walking | - Walking ≥150 min/week - Walking< 150 min/week | GIS (ArcGIS 9.2) | *Total of three variables*  **(a)** Recreational amenities (gym facilities) availability/density, **(b)** Recreational facilities (sport center) proximity, and **(c)** District income. | Logistic analysis examining association between BE variables & achieving ≥150 min/week leisure walking | Significant positive associations between recreational facilities density- (aOR=1.89; 95%CI; 1.21–2.97) & recreational facilities proximity- middle tertile (aOR=2.26; 95%CI; 1.04–2.49) and district income-highest tertile (aOR= 2.54; 95%CI; 1.48–4.37) & achieving ≥150 min/week leisure walking | [43] |
| IPAQ-LF, transport walking | - Walking ≥10 min/week - Walking <10 min/week. | GIS using a 500-m radius buffer | *Total of ten variables*  **(a)** Traffic safety, **(b)** Residential density, **(c)** Commercial density, **(d)** Street density, **(e)** Connectivity-4-way intersections proportions, **(f)** Land slope, **(g)** Bike path density, **(h)** Bus stop proximity, **(i)** Bike path proximity, and **(j)** District income | Logistic analysis examining association between each BE variable & achieving ≥10 min/week transport walking or cycling | Significant positive associations between residential density- middle tertile (aOR=1.25; 95%CI; 1.02–1.53) & commercial density- middle tertile (aOR=1.47; 95%CI; 1.13–1.91) and achieving ≥10 min/week transport walking. | [21] |
| IPAQ-LF, transport cycling | - Cycling ≥10 min/week - Cycling <10 min/week. |  |  |  | Significant negative associations between district income, highest tertile (aOR=0.26; 95%CI; 0.08–0.81), traffic safety (aOR=0.27; 95%CI; 0.09–0.84), and residential density, middle tertile (aOR=0.53; 95%CI; 0.34–0.83), and achieving ≥10 min/week transport cycling. |  |
| IPAQ-LF leisure walking | - Walking ≥150 min/week - Walking <150 min/week | NEWS- America | *Total of six variables*  **(a)**Aesthetics, **(b)** Safety to walk, **(c)** Traffic safety, **(d)** Crime safety, **(e)** Proximity to parks, and **(f)**Transit stops access/proximity. | Hurdle regression models examining association between NEWS scores and **a)** leisure walking participation, and **b)** leisure walking duration. | - Perceived aesthetics (only among people with low SES) was positively associated with leisure walking participation [exp (b)= 2.23, exp 95%CI; 1.43-3.49], and duration [exp (b)= 1.92, exp 95%CI; 1.18-3.13]. - Significant positive association between traffic safety and leisure walking duration [exp (b)= 1.33, exp 95%CI; 1.18-3.13]. | [36] |
| IPAQ-LF leisure walking | - Walking > 90 min/week - Walking ≤ 90 min/week | NEWS- China | *Total of four variables*  **(a)** Services accessibility [including markets, parks, bus stops ..etc], **(b)** Aesthetics [including trees, sanitation, lighting, roads condition, natural sights, interesting things & places for walking], **(c)** Traffic safety, and **(d)** Crime safety. | Logistic analysis examining association between NEWS scores & reaching > 90 min/week leisure walking | Significant positive association between service accessibility and reaching > 90 min/week leisure walking (aOR =1.062, 95%CI; 1.016-1.110) | [51] |
| IPAQ-LF transport walking |  |  |  | Logistic analysis examining association between NEWS scores and reaching > 90 min/week transport walking | Significant positive association between service accessibility and reaching > 90 min/week transport walking (aOR =1.053, 95%CI; 1.008-1.100). |  |
| IPAQ-SF combined leisure & transport walking | - Walking ≥150 min/week - Walking <150 min/week | PANES | *Total of 11 variables*  **(a)** Residential density, **(b)** Shops accessibility, **(c)** Transit stop accessibility, **(d)** Recreational amenities, **(e)** Bike path availability, **(f)** Sidewalks, **(g)** Crime safety at night, **(h)** Crime safety during the day, **(i)** Traffic safety, **(k)** Aesthetics, and **(l)** Street connectivity. | Logistic analysis examining association between PANES scores & reaching ≥ 150 min/week total walking. | - Significant positive association between perceived safety from crime at night (aOR=1.53, 95%CI; 1.02-2.35) & perceived pleasant aesthetics (aOR=1.90, 95%CI; 1.33-2.69) and reaching ≥ 150 min/week total walking | [12] |
| IPAQ-SF combined leisure & transport walking | - Walking ≥150 min/week - Walking <150 min/week | PANES | *Total of three variables*  **(a)** Traffic safety, **(b)** Crime safety during day, and **(c)** Crime safety at night. | Logistic analysis examining association between BE scores and reaching ≥ 150 min/week total walking | Significant positive association between perceived crime safety during the day (aOR= 5.92, 95%CI; 1.38–60.59) & at night (aOR= 6.99, 95%CI; 2.71–18.04) and reaching ≥ 150 min/week total walking. | [52] |
| IPAQ-LF, leisure walking | - PA continuous scores- (weekly frequency* PA daily duration in min) | NEWS- Brazil | *NEWS- Total of four variables*  **(a)** Proximity to services, **(b)** Traffic safety, **(c)** Crime safety, and **(d)** Accessibility (sidewalks, land slope, and bike path availability). | Logistic analysis examining association between each BE variable and each PA domain/type | No significant associations were found between any BE variable and leisure walking | [33] |
| IPAQ-LF, transport walking |  |  |  |  | Significant positive association was found between crime safety (aOR: 1.5, 95%CI; 1.0–2.1) and transport walking |  |
| IPAQ-LF, transport cycling |  |  |  |  | Significant positive association was found between proximity to services (aOR: 2.5, 95%CI; 1.2–5.2) and transport cycling. |  |
| IPAQ-LF, leisure walking | Self-reported frequency and time spent in each domain | NEWS- Brazil | *Total of 2 variables*  (a) Crime safety during the day, and (b) Crime safety at night | Poisson regression analysis examining associations between BE variables and leisure or travel walking. | No significant associations were found between any BE variable and leisure walking | [48] |
| IPAQ-LF, transport walking |  |  |  |  | Only significant negative association between feeling unsafe from crime at night and transport walking (aPR: 0.73, 95%CI; 0.57–0.94) |  |
| IPAQ-LF, transport walking | - Walking ≥150 min/week - Walking <150 min/week | NEWS- Brazil | *Total of four variables*  **(a)** Sidewalks, **(b)** Traffic safety, **(c)** Safety to walk/cycle during day, and **(d)** Safety to walk/cycle at night | Poisson regression examining association between BE scores and transport cycling or reaching ≥ 150 min/week transport walking | No significant associations were found between any BE variable and achieving ≥150 min/week transport walking | [34] |
| IPAQ-LF, transport cycling | - Cycling - Not cycling |  |  |  | No significant associations were found between any BE variable and transport cycling. |  |
| IPAQ-LF, leisure walking | - Walking ≥150 min/week - Walking <150 min/week | - GIS - Census data | *Total of two variables*  **(a)** walkability index [estimated utilizing three indicators, land-use mix, housing density, connectivity], and (**b)** District income | Logistic analysis examining association between each BE variable & achieving ≥150 min/week leisure walking | No association was found between any of the BE variables and achieving ≥150 min/week leisure walking | [47] |
| IPAQ-LF, transport walking |  |  |  | Logistic analysis examining association between each BE variable & achieving ≥150 min/week transport walking | An independent positive association between walkability and achieving ≥150 min/week travel walking (aOR=2.10, 95%CI; 1.31- 3.37). |  |
| SIMPAQ combined leisure & travel walking | Combined time spent on total walking in min/day. | NEWS-Africa | *Total of nine variables*  **(a)** Services proximity, **(b)** Recreation amenities existence, **(c)** Sidewalks, **(d)** Bike path, **(e)** Aesthetics, **(f)** Traffic safety, **(g)** Crime safety at night, **(h)** Crime safety during the day, and **(i)** Safety to walk. | Multivariate analysis examining association between NEWS scores & combined total time of walking. | 13% of the variance in time spent on total walking was elaborated by services accessibility and the availability of sidewalks. | [41] |

PA= physical activity; BE= built environment; IPAQ-LF= International Physical Activity Questionnaire- long form; NEWS= Neighbourhood Environment Walkability Scale; SIMPAQ= Simple Physical Activity Questionnaire; PANES= Physical Activity Neighbourhood Environment Scale; BMI= body mass index; MVPA= moderate to vigorous physical activity, UGS= urban green spaces; SES= socioeconomic status; GIS= geographic information system

**S4 Appendix. Classification of data according to PA domains- Total physical activity**

| **Total PA** | | **Built environment** | | **Analysis** | **Main findings** | **Reference** |
| --- | --- | --- | --- | --- | --- | --- |
| **PA tool** | **PA variables** | **BE tool** | **BE variables** |  |  |  |
| Tailored, self-reported PA questionnaire | - ≥150 min/week MVPA - < 150 min/week MVPA | Urbanicity composite measure, | *Total of three variables*  (a) high urbanicity level, (b) medium urbanicity level, and (c) low urbanicity level | Logistic analysis examining association between each urbanicity level & achieving < 150 min/week MVPA | The odds of achieving < 150 min/week MVPA showed significant increase with urbanicity amongst both males (medium urbanicity- aOR=1.7, 95%CI; 1.2- 2.5; high urbanicity- aOR=3.2, 95%CI; 2.5- 4.2) and females (high urbanicity- aOR=4.2, 95%CI; 3.0- 5.7). | [58] |
| Accelerometer (Actigraph GT3X) | - ≥150 min/week MVPA - < 150 min/week MVPA | NEWS- America | *Total of six variables*  **(a)**Aesthetics, **(b)** Pedestrian infrastructure/sidewalks, **(c)** Traffic safety, **(d)** Crime safety, **(e)** Parks proximity, and **(f)** Transit stops proximity. | Multiple regression analysis examining association between BE scores & reaching ≥ 150 min/week MVPA | - Significant positive association between perceived crime safety-among males- (aOR=1.29, 95%CI; 1.07- 1.57) & parks proximity (aOR= 1.12, 95%CI; 1.01- 1.24) and reaching ≥ 150 min/week MVPA. - Significant negative association between transit stops proximity and reaching ≥ 150 min/week MVPA (aOR= 0.81, 95%CI; 0.72- 0.90). | [53] |
| IPAQ-SF | - ≥150 min/week MVPA   < 150 min/week MVPA | Census data | Urban vs rural settings | Logistic analysis examining association between urbanicity achieving < 150 min/week MVPA | Urbanicity is significantly positively associated with achieving < 150 min/week MVPA (OR=2.5, 95%CI; 2.1–2.9). | [57] |
| IPAQ | - ≥150 min/week MVPA   < 150 min/week MVPA | Self-reported questionnaire | Urban vs rural settings | Logistic analysis examining association between urbanicity and achieving < 150 min/week MVPA | Urbanicity is significantly positively associated with achieving < 150 min/week MVPA (OR=1.56, 95%CI; 1.43–1.71). | [56] |
| Accelerometer (ActiGraph GT3X) | - Achieving 6167–9642 counts/min - Achieving ≥9643 counts/min | GIS (ArcGIS 9.3), using radial buffers of 500, 1000, and 1600 m | *Total of two variables*  **(a)** Public services proximity [police station, health centres, open areas], and **(b)** Transit stops proximity | Multiple regression analysis examining association between BE variables & achieving 6167–9642 & ≥9643 counts/min. | Only community centers proximity within 1600-m buffers was significantly negatively associated with achieving ≥9643 counts/min (β= -69.30, 95%CI; − 134.92 to − 3.70). | [54] |
| IPAQ-SF | - ≥150 min/week MPA (*excluding walking*) - < 150 min/week MPA (*excluding walking*) | PANES | *Total of 11 variables*  **(a)** Residential density, **(b)** Shops accessibility/proximity, **(c)** Transit stop accessibility, **(d)** Recreational amenities, **(e)** Bike path availability, **(f)** Sidewalks availability, **(g)** Crime safety at night, **(h)** Crime safety during the day, **(i)** Traffic safety, **(j)** Aesthetics, and **(k)** Street connectivity. | Logistic analysis examining association between PANES scores & reaching ≥ 150 min/week MPA (*excluding walking*) | - Significant positive association between perceived transit stop accessibility and reaching ≥ 150 min/week MPA (*excluding walking*) (aOR= 1.41, CI; 1.05–1.90). - Significant unexpected negative association between perceived traffic safety (traffic not problem) (aOR= 0.45, 95%CI; 0.25– 0.81) & perceived lack of four-way intersection (aOR=0.72, 95%CI; 0.53–0.97) and reaching ≥ 150 min/week MPA (*excluding walking*). | [12] |
| Accelerometer | - ≥150 min/week MVPA - < 150 min/week MVPA | PANES | *Total of three variables*  **(a)** Traffic safety, **(b)** Crime safety during day, and **(c)** Crime safety at night. | Logistic analysis examining association between BE scores and achieving ≥ 150 min/week MVPA | - Significant positive association between perceived crime safety at night (aOR=1.68, 95%CI; 1.07–3.64) and achieving ≥ 150 min/week MVPA - Significant unexpected negative association between perceived crime safety during day and achieving ≥ 150 min/week MVPA (aOR=0.34, 95%CI; 0.06–0.91). | [52] |
| IPAQ-LF | - ≥150 min/week total PA - < 150 min/week total PA | NEWS- Brazil | *NEWS- Total of four variables*  **(a)** Proximity to services, **(b)** Traffic safety, **(c)** Crime safety, and **(d)** Accessibility (sidewalks, land slope, and bike path availability). | Logistic analysis examining association between each BE variable and achieving ≥ 150 min/week total PA. | Significant positive associations were found between accessibility (aOR: 1.4, 95%CI; 1.0–1.8), and crime safety (aOR: 1.3, 95%CI; 1.0–1.7), and achieving ≥ 150 min/week total PA. | [33] |
| IPAQ-SF | - ≥150 min/week MVPA - < 150 min/week MVPA | Tailored, self-reported BE perceptions questionnaire | *Total of two variables*  **(a)** Safety to cycle, and **(b)** Recreational amenities proximity. | Logistic analysis examining association between BE scores and achieving < 150 min/week MVPA | Significant positive unexpected association between perceived safety for cycling and achieving < 150 min/week MVPA (aOR=1.50, 95%CI; 1.05–2.16). | [55] |

PA= physical activity; BE= built environment; IPAQ-LF= International Physical Activity Questionnaire- long form; NEWS= Neighbourhood Environment Walkability Scale; SIMPAQ= Simple Physical Activity Questionnaire; PANES= Physical Activity Neighbourhood Environment Scale; BMI= body mass index; MVPA= moderate to vigorous physical activity, UGS= urban green spaces; SES= socioeconomic status; GIS= geographic information system
