# Supplementary material for: Built environment correlates of physical activity in low- and middle-income countries: A systematic review
Source: PLoS One. 2020 Mar 17;15(3):e0230454. doi: 10.1371/journal.pone.0230454 (PMC7077823; doi:10.1371/journal.pone.0230454)
Supplement: S5 Appendix — (DOCX) [file pone.0230454.s005.docx]

**S5 Appendix. Quality assessment of the included studies**

| **Author, year** | **Study design (cross-sectional/ case study=1, longitudinal/quasi design=2)** | **Reported reliable response rate (≥80%), (weight=1)** | **Method of PA measurement (weight: subjective=0.5, objective=1)** | **Method of BE measurement (weight: subjective=0.5, objective=1)** | **Stratification of recruitment areas by suitable environmental characteristics to maintain generalizability (weight=1)** | **Controlling for sociodemogr-aphic confounders (weight: 1)** | **Calculation of confidence intervals for main results (weight: 1)** | **Assessment of statistical significance (*p* value) (weight: 1)** | **Total score (maximum of 9)** |
| --- | --- | --- | --- | --- | --- | --- | --- | --- | --- |
| Adlakha et al, 2017 | cross-sectional | Not reported | Subjective | Subjective | Yes | Yes | Yes | No | 5 |
| Adlakha et al, 2018 | cross-sectional | Not reported | Subjective | Subjective | Yes | Yes | Yes | Yes | 6 |
| Akpinar et al, 2016 | Case study | Not reported | Subjective | Subjective | Yes | Yes | Yes | Yes | 6 |
| Allender et al, 2010 | cross-sectional | Yes (97%) | Subjective | Objective | No | Yes | Yes | Yes | 6.5 |
| Amorim et al, 2010 | cross-sectional | Yes (90.7%) | Subjective | Subjective | Yes | Yes | Yes | Yes | 7 |
| Cervero et al, 2009 | Cross-sectional | No (66.7%) | Subjective | Objective | Yes | No | Yes | Yes | 5.5 |
| Chen et al, 2017 | Case study | Yes (96.7%) | Subjective | Subjective | No | No | Yes | Yes | 5 |
| Cunningham-Myrie et al, 2015 | cross-sectional | Not reported | Subjective | Subjective & objective | No | Yes | Yes | Yes | 5.5 |
| Florindo et al, 2017 | cross-sectional | Not reported | Subjective | Objective | Yes | Yes | Yes | Yes | 6.5 |
| Giehl et al,2016 | cross-sectional | Yes (89.2%) | Subjective | Objective | Yes | Yes | Yes | Yes | 7.5 |
| Gómez et al, 2010a | cross-sectional | No (67.8%) | Subjective | Subjective & objective | Yes | Yes | Yes | Yes | 6.5 |
| Gomez et al, 2010b | cross-sectional | No (66%) | Subjective | Objective | Yes | Yes | Yes | Yes | 6.5 |
| Gomes et al, 2011 | cross-sectional | No (<70%) | Subjective | Subjective | Yes | Yes | Yes | Yes | 6 |
| Gul et al, 2018 | Cross-sectional | Not reported | Subjective | Objective | Yes | No | No | Yes | 4.5 |
| Hallal et al, 2010 | Cross-sectional | No (56%) | Subjective | Subjective | Yes | Yes | Yes | No | 5 |
| Hino et al, 2011 | Cross-sectional | Yes (93.2%) | Subjective | Objective | Yes | Yes | Yes | No | 6.5 |
| Hino et al, 2014 | Cross-sectional | Not reported | Subjective | Objective | Yes | Yes | Yes | Yes | 6.5 |
| Jaime et al, 2011 | Cross-sectional | Not reported | Subjective | Objective | No | Yes | No | Yes | 4.5 |
| Jáuregui et al, 2016 | Cross-sectional | Not reported | Objective | Subjective | Yes | Yes | Yes | Yes | 6.5 |
| Jáuregui et al, 2017 | Cross-sectional | Not reported | Subjective | Subjective | Yes | Yes | Yes | No | 5 |
| Jia et al, 2014 | Cross-sectional | Yes (84.9%) | Subjective | Subjective | Yes | Yes | Yes | Yes | 7 |
| Katulanda et al, 2012 | Cross-sectional | Yes (89.7 %) | Subjective | Objective | No | No | Yes | Yes | 5.5 |
| Koyanagi et al, 2018 | Cross-sectional | Yes (98.5%) | Subjective | Subjective | Yes | Yes | Yes | Yes | 7 |
| Malambo et al, 2018 | Cross-sectional | Not reported | Objective | Objective | No | Yes | Yes | Yes | 6 |
| Oyeyemi et al, 2011 | Cross-sectional | Yes (85.8%) | Subjective | Subjective | No | Yes | Yes | Yes | 6 |
| Oyeyemi et al, 2012 | Cross-sectional | Not reported | Subjective & objective | Subjective | Yes | Yes | Yes | Yes | 6.5 |
| Parra et al, 2011 | Cross-sectional | Not reported | Subjective | Subjective | Yes | Yes | Yes | Yes | 6 |
| Rech et al, 2012 | Cross-sectional | Not reported | Subjective | Subjective | Yes | Yes | Yes | Yes | 6 |
| Rech et al, 2014 | Cross-sectional | Not reported | Subjective | Subjective | Yes | Yes | Yes | Yes | 6 |
| Reis et al, 2013a | Cross-sectional | No (<70%) | Subjective | Subjective | Yes | Yes | Yes | No | 5 |
| Reis et al, 2013b | Cross-sectional | No (66.4%) | Subjective | Objective | Yes | Yes | Yes | Yes | 6.5 |
| Trude et al, 2016 | Cross-sectional | Not reported | Subjective | Subjective | Yes | Yes | Yes | Yes | 6 |
| Vancampfort et al, 2019 | Cross-sectional | Not reported | Subjective | Subjective | No | Yes | No | Yes | 4 |

Higher scorings indicate better quality: ≤4 (low quality), 4.1-5.9 (intermediate quality), 6-6.9 (high quality), and ≥7 (very high quality).
