# Supplementary material for: Built environment correlates of physical activity in low- and middle-income countries: A systematic review
Source: PLoS One. 2020 Mar 17;15(3):e0230454. doi: 10.1371/journal.pone.0230454 (PMC7077823; doi:10.1371/journal.pone.0230454)
Supplement: S1 Table — (DOCX) [file pone.0230454.s006.docx]

**S1 Table. Numerical distribution and characteristics of the included studies**

| **Study category** | **Number of studies n (%)** |
| --- | --- |
| **Country** | |
| Brazil | 14 (43%) |
| India | 3 (9%) |
| Mexico | 2 (6%) |
| China | 2 (6%) |
| Turkey | 1 (3%) |
| Jamaica | 1 (3%) |
| Colombia | 3 (9%) |
| Pakistan | 1 (3%) |
| Sir Lanka | 1 (3%) |
| Nigeria | 2 (6%) |
| South Africa | 1 (3%) |
| Uganda | 1 (3%) |
| Mixed LMICs | 1 (3%) |
| **Study Design** | |
| Cross-sectional | 31 (94%) |
| Case study | 2 (6%) |
| **Year of publication** | |
| 2009-2012 | 14 (43%) |
| 2013-2015 | 6 (18%) |
| 2016-2019 | 13 (39%) |
| **Study quality** | |
| Low-quality (score ≤4) | 1 (3%) |
| Medium-quality (4.1-5.9) | 10 (30%) |
| High-quality (score 6-6.9) | 18 (55%) |
| Very high-quality (score ≥7) | 4 (12%) |
| **PA domains*** | |
| Leisure PA | 16 (49%) |
| Travel PA | 5 (15%) |
| Leisure walking | 10 (30%) |
| Transport walking | 8 (24%) |
| Total walking | 4 (12%) |
| Transport cycling | 4 (12%) |
| Total PA | 9 (27%) |
| **BE variables*** | |
| Land-use mix diversity | 3 (9%) |
| Land-use mix access | 2 (6%) |
| Residential density | 6 (18%) |
| Commercial density | 1 (3%) |
| Street density | 3 (9%) |
| Park density | 3 (9%) |
| Recreational amenities availability | 8 (24%) |
| Recreational facilities proximity | 9 (27%) |
| Transit stops proximity | 9 (27%) |
| Services proximity | 3 (9%) |
| Squares proximity | 1 (3%) |
| Aesthetics | 11 (33%) |
| Safety to cycle/walk | 8 (24%) |
| Walkability | 1 (3%) |
| Street connectivity | 7 (21%) |
| Sidewalks | 10 (30%) |
| Terrain slope | 4 (12%) |
| Paved streets | 1 (3%) |
| Bike path availability | 5 (15%) |
| Bike path proximity | 3 (9%) |
| Crime safety | 15 (46%) |
| Traffic safety | 15 (46%) |
| District income | 4 (12%) |
| Urbanicity | 3 (9%) |

*The percentages are not additive as some studies addressed more than one PA domain and BE attribute.
